# Nutritional Strategies for Recovery–Adaptation Coupling After Exercise: From Muscle Damage to Performance Remodeling

**DOI:** 10.3390/nu18152523

**Published:** 2026-08-04

**Authors:** Dan Cristian Mănescu, Giuseppe Cerullo, Cristian Petri, Costin Petcu, Radu Bidiugan, Andreea Voinea, Simona Bidiugan, Alexandra Reta Iacobini, Andrei Dragomir, Alexandru Adrian Gavrilă, Răzvan Alexandrescu, Cristian Băltărețu, Răzvan Liviu Petre

**Affiliations:** 1 Department of Physical Education and Sport, Bucharest University of Economic Sciences, 010374 Bucharest, Romania; dan.manescu@defs.ase.ro (D.C.M.); andreea.voinea@defs.ase.ro (A.V.); 2 Department of Biomedical Sciences, University of Padova, 35131 Padova, Italy; giuseppe.cerullo@unipd.it; 3 Department of Sports and Computer Science, Section of Physical Education and Sports, Universidad Pablo de Olavide, 41013 Seville, Spain; cpetri@ext.acffiorentina.it; 4 Faculty of Medicine, Carol Davila University of Medicine and Pharmacy, 050474 Bucharest, Romania; 5 National Institute for Sport Research, 022103 Bucharest, Romania; bidiugan@sportscience.ro (R.B.); catanoiu@sportscience.ro (S.B.); andrei.dragomir@sportscience.ro (A.D.); 6 Department of Physical Education and Sports, Faculty of Physical Education and Sport, Spiru Haret University, 030045 Bucharest, Romania; alexandra.iacobini@spiruharet.ro; 7 Department of Management Information Systems, Bucharest University of Economic Studies, 010374 Bucharest, Romania; alexandru.gavrila@cig.ase.ro; 8 Faculty of Physical Education and Sports, National University of Physical Education and Sports, 060057 Bucharest, Romania; razvan.petre@unefs.ro; 9 Romanian Bodybuilding and Fitness Federation (FRCF), 030167 Bucharest, Romania

**Keywords:** sports nutrition, muscle recovery, exercise-induced muscle damage, delayed-onset muscle soreness, inflammation resolution, oxidative stress, muscle protein synthesis, polyphenols, micronutrients, recovery monitoring, precision nutrition

## Abstract

**Background/Objectives:** Recovery nutrition must restore near-term readiness without indiscriminately suppressing biological signals that contribute to repair and training adaptation. This review evaluates recovery–adaptation coupling (RAC) as a research framework and clarifies its contribution relative to established recovery, nutrient-periodization, and athlete-monitoring models. **Methods:** Targeted narrative searches of PubMed/MEDLINE, Scopus, and Web of Science were supplemented by Google Scholar citation tracking and backward and forward screening. Peer-reviewed English-language literature available through 31 May 2026 was considered. Human athlete studies, randomized trials, systematic reviews, meta-analyses, consensus statements, and position stands were prioritized; mechanistic evidence was used to explain pathways rather than to support stand-alone performance recommendations. The final cited corpus comprised 130 records. No formal risk-of-bias tool, certainty grading, PRISMA denominator, or quantitative pooling was used. Claims were instead identified as established practice (EP), context-dependent evidence (CDE), mechanistic rationale (MR), or RAC hypothesis (RH). **Results:** The most consistent applied support concerns adequate energy availability, distributed high-quality protein, carbohydrate restoration when recovery windows are short, and individualized fluid and sodium replacement. Evidence for polyphenol-rich products, curcumin, omega-3 fatty acids, and creatine is context- and product-dependent. Collagen or gelatin evidence is mainly mechanistic or pilot-level, while RAC recovery-pattern categories and multimodal monitoring rules remain unvalidated hypotheses. RAC differs from existing frameworks by jointly specifying the next athletic demand, dominant recovery bottleneck, possible adaptive cost of intervention, and response-verification plan. **Conclusions:** RAC should presently be interpreted as an evidence-organization and hypothesis-generation architecture, not as a validated predictive, diagnostic, or treatment algorithm. Prospective comparative studies are required before RAC-specific decision rules can guide individualized practice.

## 1. Introduction

Recovery is an active interval between exercise exposures in which damaged contractile and extracellular-matrix structures are repaired, inflammatory and redox processes are coordinated, substrates are restored, and cellular signals are translated into future performance capacity [1,2,3,4]. High-intensity, eccentric, repeated-sprint, endurance, and contact-sport loads can produce delayed-onset muscle soreness (DOMS), transient strength loss, reduced power, glycogen depletion, autonomic disturbance, and perceived fatigue; when appropriately scaled and resolved, the same perturbations can also initiate remodeling and adaptation [5,6,7,8,9]. Consequently, the nutritional priority varies with the next demand: congested competition or repeated same-day sessions may require rapid functional restoration, whereas adaptation-oriented training may call for preserving redox and inflammatory signaling.

Most practical discussions of recovery nutrition remain product-centered: protein, carbohydrate, antioxidants, creatine, polyphenols, omega-3 fatty acids, nitrates, collagen, or micronutrients. This catalog approach is useful but incomplete. Athletes experience recovery across the interval between exercise exposures—including immediate, between-session, and overnight periods—as a pattern of soreness, force loss, substrate depletion, sleep disruption, psychological readiness, connective-tissue strain, and next-session demands. RAC therefore does not propose a new supplement hierarchy; it couples the dominant recovery bottleneck, the urgency and biological purpose of the next demand, the adaptive signal that may be modified, and the monitoring pattern used to confirm benefit. This distinguishes RAC from general recovery checklists and from fuel-periodization models [3,4,10,11].

[RH] This review proposes RAC as an integrative model for organizing nutrition across the interval between exercise exposures. Its proposed decision sequence is to define the next athletic demand, identify the dominant recovery bottleneck, consider the adaptive signal that an intervention might modify, and pre-specify the marker pattern used to judge response. RAC is therefore operational only as a research architecture and hypothesis-generating model; it is not a validated treatment algorithm [3,4,6,10,11,12,13,14,15,16].The comparison in Table 1 makes the claimed distinction from adjacent frameworks explicit.

The review has three focused objectives (O), each mapped to the subsequent synthesis:

**O1**. Synthesize the biological processes and nutritional strategies relevant to symptom recovery, tissue recovery, functional recovery, and adaptive readiness (Section 3, Section 4, Section 5, Section 6, Section 7 and Section 8).
**O2**. Distinguish established practice from context-dependent human evidence, mechanistic rationale, and RAC-generated hypotheses, while defining RAC relative to adjacent frameworks (Section 2, Section 3, Section 4, Section 5, Section 6, Section 7 and Section 8 and Table 1).
**O3**. Specify research-facing monitoring procedures, professional boundaries, and directly testable RAC hypotheses rather than presenting an unvalidated clinical or performance algorithm (Section 9, Section 10, Section 11 and Section 12).

## 2. Review Approach and Methodological Framework

### 2.1. Search Strategy and Source Identification

Relevant literature was identified through targeted searches of PubMed/MEDLINE, Scopus, and Web of Science, supplemented by Google Scholar citation tracking and backward and forward screening of key reviews, meta-analyses, consensus statements, and position stands. Coverage extended from database inception through 31 May 2026. Only peer-reviewed articles in English were considered. Conference abstracts, duplicate publications, non-peer-reviewed sources, and records lacking sufficient methodological or outcome detail were de-prioritized. The original searches were iterative, and contemporaneous day-level execution logs and complete hit counts were not retained. To avoid retroactively inventing precision, Supplementary File S2 identifies this limitation and provides database-ready Boolean strings, fields, filters, and concept blocks that reconstruct the reported search logic for reproducibility rather than presenting them as a prospectively archived systematic-review log.

The search concepts covered exercise-induced muscle damage, delayed-onset muscle soreness, inflammation and resolution, oxidative and redox signaling, muscle protein synthesis, protein dose and timing, glycogen resynthesis, carbohydrate availability, hydration and electrolytes, energy availability and RED-S, omega-3 fatty acids, polyphenol-rich foods, tart cherry, pomegranate, blueberry, curcumin, creatine, dietary nitrate, collagen or gelatin, vitamin C, vitamin D, iron, magnesium, sleep, heart-rate variability, wearables, athlete monitoring, training load, sex-specific reporting, and gut tolerance. Database-specific syntax and field restrictions are listed in Supplementary Section S2.2.

The search process followed three iterative passes. Pass 1 identified consensus statements, position stands, and high-level reviews defining accepted principles of sports nutrition, recovery, monitoring, and supplementation. Pass 2 identified randomized trials, controlled human studies, systematic reviews, and meta-analyses for specific nutrients, foods, and compounds. Pass 3 added mechanistic, connective-tissue, redox, immune, microbiome, and monitoring studies that clarified plausible pathways but were not used alone to justify applied sport-performance recommendations. Google Scholar was used for citation chaining rather than as a source of a countable search denominator.

The final cited evidence base comprised 130 records. Because the review was narrative, iterative, and citation-led rather than protocol-registered and PRISMA-driven, a reliable denominator for all records initially viewed, duplicated, excluded, or discovered through citation chasing cannot be reconstructed. Supplementary Figure S1 therefore presents a transparent narrative selection pathway without fabricated counts, and Supplementary Table S1 provides a complete inventory of all 130 cited records and their role in the synthesis.

### 2.2. Eligibility, De-Prioritization, and Evidence Weighting

Evidence was eligible for interpretive use when it addressed at least one of four domains: (1) biological recovery processes after exercise; (2) nutrition, functional foods, or supplements affecting recovery- or adaptation-related mechanisms; (3) applied outcomes such as strength, power, repeated-sprint ability, endurance capacity, soreness, fatigue, or readiness; or (4) monitoring variables used to interpret recovery status. A claim, rather than an entire article, could map to more than one domain.

Operational prioritization was defined as follows. Direct transferability required trained or athletic participants or an exercise model closely matching the proposed use. An adequate comparator was a concurrent placebo, usual-practice, matched-diet, or within-participant condition exposed to the same exercise and measurement schedule. Product characterization required a stated formulation, dose, timing, and duration. Functional alignment required an outcome relevant to the proposed application and a recovery window that overlapped the next demand. Studies with fewer than 10 participants per arm were treated as exploratory unless supported by larger or replicated evidence. Mechanistic studies in healthy adults, clinical populations, cells, animals, or engineered tissue were used only to explain pathways and not as stand-alone support for performance, injury-prevention, rehabilitation, or return-to-play claims.

Records were de-prioritized when they relied exclusively on untrained populations without a clear transfer argument, poorly characterized products, non-specific wellness endpoints, isolated biomarkers without functional interpretation, very short or mismatched recovery windows, absent concurrent comparators, or conclusions exceeding the design. Interpretation also considered baseline nutritional status, total energy intake, training status, sex-specific reporting, adverse-event reporting, and proximity to the next training or competition demand.

When findings conflicted, interpretation favored trained or athletic samples, clearly characterized interventions, adequate concurrent comparators, functional outcomes, and recovery windows matching the proposed scenario. Null and inconsistent findings were retained when they constrained a best-use context. The original narrative selection was not performed by a fixed two-reviewer systematic-screening team, and no prospective disagreement log or inter-rater statistic exists; this absence is now reported explicitly rather than reconstructed. The revision adds a post hoc claim-level inventory of the complete cited corpus, but that audit is not represented as independent duplicate screening.

Six of the 130 references (4.6%; references [12,13,14,15,19,31]) include one or more members of the current author group. These papers are used only for conceptual comparison, framework positioning, or biomarker-organization context; they are not treated as empirical validation of RAC or as evidence of intervention efficacy. Independent consensus statements, systematic reviews, mechanistic literature, and human trials provide the substantive support for applied claims.

### 2.3. Synthesis Logic and Transparency Limits

Recovery outcomes were grouped at claim level into four non-exclusive domains. Symptom recovery comprised self-reported DOMS, fatigue, mood, appetite, sleep quality, and perceived readiness. Tissue recovery comprised structural, muscle-damage, inflammatory, redox, collagen-turnover, hydration, and micronutrient-status markers. Functional recovery comprised restoration of force, power, sprint ability, repeated-effort output, endurance capacity, and sport-specific performance. Adaptive readiness comprised chronic remodeling, signaling, and training-response outcomes relevant to future capacity. For example, a tart-cherry trial could contribute simultaneously to symptom and functional domains, whereas a gelatin-PINP study contributes primarily to tissue-mechanistic interpretation.

Claim provenance was coded with four labels. EP (established practice) denotes guidance supported by consensus or position statements and replicated human evidence in the relevant population. CDE (context-dependent evidence) denotes human evidence that is heterogeneous, product-specific, population-limited, or inconsistent. MR (mechanistic rationale) denotes biological plausibility or surrogate evidence without demonstrated applied benefit. RH (RAC hypothesis) denotes an author-generated linkage, threshold, category, or prediction not prospectively validated as part of RAC. These labels describe the source and maturity of a claim; they are not GRADE ratings, formal certainty judgments, or numerical rankings. Representative rules and examples are reproduced in Supplementary Section S2.5.

The synthesis followed a target-to-strategy-to-context sequence: identify the dominant bottleneck, define the purpose and time horizon of the next demand, evaluate the plausible adaptive consequence of an intervention, and specify the marker pattern that could support continuation, modification, or rejection of the hypothesis. EP, CDE, MR, and RH labels are applied at paragraph or table-row level in Section 3, Section 4, Section 5, Section 6, Section 7, Section 8, Section 9, Section 10 and Section 11 so that consensus practice is not blended with RAC-specific extrapolation.

Narrative evidence-selection pathway: targeted database and citation searches → relevance screening against recovery biology, nutritional strategy, applied outcome, adaptive consequence, or monitoring domain → transferability and design prioritization → claim-level mapping to outcome domain and provenance status → audit of the final 130-record cited corpus. Supplementary Figure S1 depicts this sequence and its transparency limit.

No formal risk-of-bias scoring, certainty grading, PRISMA flow diagram, or pooled quantitative synthesis was performed because the review was not designed as an intervention-specific systematic review. Accordingly, comparative descriptors such as “high,” “moderate,” or “low” evidence have been removed from the revised tables. EP, CDE, MR, and RH identify claim provenance only and must not be interpreted as formal certainty grades. RAC remains a conceptual architecture for organizing evidence and generating hypotheses, not a validated predictive model or a substitute for individualized clinical, dietetic, or performance judgment.

## 3. Exercise-Induced Muscle Damage and the Biology of Recovery

Exercise-induced muscle damage (EIMD) is a coordinated continuum in which mechanical disruption, metabolic perturbation, oxidative stress, inflammatory signaling, and molecular remodeling evolve over different time courses [32,33,34,35,36,37]. Classical markers such as creatine kinase, lactate dehydrogenase, and myoglobin are useful but indirect and highly variable, whereas structural markers may provide greater tissue specificity [37].

Inflammation and redox activity are not uniformly deleterious: immune cells support debris clearance, satellite-cell activation, and tissue remodeling, while reactive oxygen and nitrogen species also participate in mitochondrial biogenesis, antioxidant defense, and glucose transport [38,39,40,41,42,43,44,45]. The practical objective is therefore modulation rather than blanket suppression.

Because soreness, force loss, and circulating biomarkers recover on different timelines, nutritional decisions should be matched to the dominant functional bottleneck—structural damage, substrate depletion, connective-tissue strain, or systemic fatigue—and evaluated through convergent markers [1,2,37]. The main processes and targets are summarized in Table 2.

## 4. The Recovery–Adaptation Coupling Framework

The four RAC targets are overlapping analytic lenses rather than independent, exhaustive, or causally ordered biological stages. Damage attenuation means limiting avoidable structural disruption or performance loss without assuming that all damage signals should be eliminated. Inflammation resolution means supporting the transition from activation to homeostasis rather than indiscriminate inhibition. Anabolic remodeling means restoring energy and amino-acid substrates and supporting myofibrillar and extracellular-matrix turnover. Adaptive-signal preservation means avoiding unnecessary suppression of redox, inflammatory, metabolic, or mechanical signals when longer-term adaptation is the priority. A single intervention can influence several lenses simultaneously; therefore, the model does not claim construct independence.

[CDE] In a team-sport athlete competing again within 24 h, glycogen depletion and sweat loss may make carbohydrate, protein, sodium, and fluid replacement the dominant priorities, verified by body-mass recovery and repeated-effort performance [18,64,65,66,67,68,69,70,71]. [CDE/MR] In a non-urgent endurance microcycle, adequate energy and protein with periodized carbohydrate may be preferable to routine high-dose antioxidant or anti-inflammatory supplementation [10,11,41,42,43,44]. [MR] During tendon rehabilitation, collagen or gelatin with vitamin C is biologically plausible only when coupled to progressive loading; evidence for accelerated return to play remains insufficient [86,87,88].

**Figure 1:**
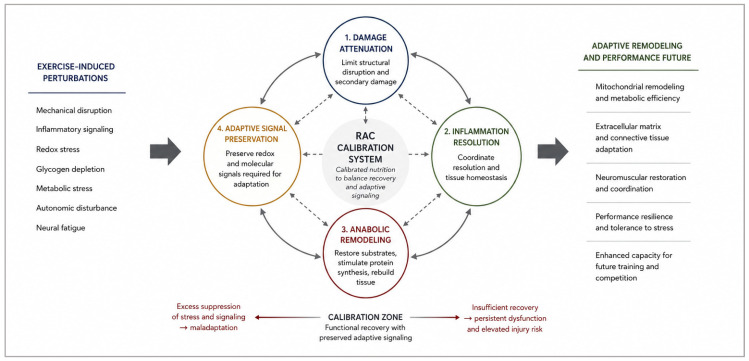
Recovery–adaptation coupling (RAC) framework with explicit provenance coding. The four targets are overlapping analytic lenses, not independent, exhaustive, or causally ordered stages. Solid arrows indicate broadly accepted biological or consensus-consistent relationships; dashed arrows indicate RAC-generated theoretical links requiring prospective validation; feedback loops represent reassessment rather than causal proof. RAC = recovery–adaptation coupling.

Figure 1 separates evidence-supported biological relations from RAC-generated links. Solid arrows denote broadly accepted process relations or consensus-consistent management links; they do not prove that RAC itself is effective. Dashed arrows denote theoretical inferences or author-generated assumptions requiring prospective testing, including adaptive-signal trade-offs, phenotype matching, and monitoring-confirmed escalation. Feedback loops denote reassessment, not causal proof.

## 5. Macronutrient Strategies for Muscle Recovery

### 5.1. Protein Quantity, Quality, and Distribution

[EP] Protein is central to recovery because resistance, endurance, and damaging exercise alter muscle protein turnover [72,73,74,75,76,77,78,79,80,81,82]. The often-cited starting range of approximately 0.25–0.40 g/kg of high-quality protein per feeding, commonly 20–40 g, derives mainly from healthy adult resistance-exercise and athlete studies. It should be interpreted within total daily protein and energy intake, protein quality, meal distribution, body size, training mode, age, and gastrointestinal tolerance; older or anabolic-resistant athletes may require the upper part of the range. These values are not universal prescriptions for children, pregnancy, renal disease, acute injury, or other clinical conditions [72,73,74,75,76,77,78,79,80,81,82].

[CDE] Pre-sleep protein may be useful after late training or when a long overnight interval limits amino-acid availability. Trials using approximately 30–40 g of casein-rich protein in healthy young men have increased overnight muscle protein synthesis [83,84], but transfer to women, older athletes, other protein sources, weight-sensitive contexts, and clinical populations requires individualized interpretation. Early increases in muscle protein synthesis after severe damage may reflect repair rather than hypertrophy [85].

### 5.2. Carbohydrate Availability and Glycogen-Centered Recovery

[EP] Carbohydrate is most urgent after substantial glycogen-depleting exercise when the next glycogen-dependent session occurs within approximately 8 h. In that narrow scenario, 1.0–1.2 g/kg/h during the first 4 h is a commonly studied range [64,65,66,67,68]. It is not required when the next demanding session is more than approximately 24 h away or when glycogen demand is low. Body size, sex, habitual intake, exercise modality, total energy intake, gastrointestinal tolerance, and the magnitude of depletion should determine the final plan. Carbohydrate–protein co-ingestion is mainly practical when carbohydrate intake or appetite is limited [64,65,66,67,68].

[CDE] Carbohydrate availability can be periodized: high availability supports repeated high-intensity work and competition readiness, whereas deliberately lower availability may amplify selected endurance-training signals. The latter strategy can also reduce training quality or energy availability and should not be generalized to athletes with RED-S risk, heavy competition schedules, or inadequate baseline intake [10,11].

### 5.3. Lipid Quality, Omega-3 Fatty Acids, and Membrane-Based Recovery

[CDE] Dietary fat quality influences membrane composition and inflammatory lipid mediators. Multi-week omega-3 supplementation has altered strength loss, stiffness, or anabolic responses in selected cohorts, but the products, doses, populations, and outcomes are heterogeneous [46,47,48,49,50]. It should not be presented as an acute analgesic or as a substitute for energy sufficiency and dietary quality.

[EP] Lipid strategies should remain part of an adequate overall diet. Isolated supplements cannot compensate for low energy availability, poor protein distribution, insufficient carbohydrate, micronutrient deficiency, or inadequate sleep.

### 5.4. Integrated Macronutrient Recovery

[EP] An effective recovery meal is rarely a single-nutrient solution. Healthy adult athletes may require protein for remodeling, carbohydrate for substrate restoration, fluid and sodium for rehydration, and foods that are tolerable during travel or short recovery windows. The composition should be written as a scenario linked to the next demand rather than as a generic prescription.

[CDE] As an illustrative scenario rather than a universal dose, a 75 kg healthy adult athlete completing a hot 90 min session before evening training might combine approximately 75–90 g carbohydrate, 25–30 g high-quality protein, and sodium-containing fluid guided by measured body-mass loss [64,65,66,67,68,69,70,71]. Different body sizes, sweat rates, exercise modes, clinical conditions, and recovery intervals require adjustment. Table 3 summarizes these boundaries.

## 6. Bioactive Compounds and Functional Foods in Exercise Recovery

### 6.1. Polyphenol-Rich Foods

[CDE] Polyphenol-rich foods show product-specific rather than class-wide effects, and the cited trials are generally small. In the 14-participant randomized crossover trial by Connolly et al. [53], average post-eccentric strength loss was 22% with placebo and 4% with tart cherry, whereas elbow angle and tenderness did not differ. Bell et al. [56] randomized 16 semi-professional male soccer players and reported selected improvements after prolonged intermittent exercise. In 10 physically active women, McLeay et al. [57] observed a treatment-by-time interaction for peak isometric torque (*p* = 0.047), but not a parallel reduction in soreness. These findings indicate possible context-specific functional effects, not a universal class effect.

[CDE] Pomegranate evidence is similarly constrained: one crossover study included nine elite weightlifters and reported selected performance, soreness, and biomarker differences after a high-volume juice protocol [58]. Across tart cherry, pomegranate, and blueberry studies, products, polyphenol content, exercise models, outcomes, and timing vary markedly. Small sample sizes, selective positive outcomes, sparse precision reporting, and incomplete adverse-event collection limit generalization; functional recovery and consistency across outcomes should take precedence over isolated biomarker changes [51,52,53,54,55,56,57,58,59,98,99].

### 6.2. Curcumin, Nitrates, and Creatine

[CDE] Curcumin trials also require calibrated interpretation. Drobnic et al. [60] randomized 20 moderately active men in a pilot downhill-running trial using a phytosome providing 200 mg curcumin twice daily and reported selected pain, imaging, and inflammatory differences, but not uniform benefit across outcomes. In a 17-man crossover trial, Nicol et al. [61] used 2.5 g twice daily and reported pain reductions of approximately 1.0–1.9 cm on 10 cm scales, creatine kinase reductions of approximately 22–29%, and a 15% increase in single-leg jump performance, with wide 90% compatibility limits. Meta-analytic synthesis suggests possible DOMS benefit, but formulation, bioavailability, dose, population, and outcome selection remain important [62,63]. Short research exposures do not establish long-term safety or justify chronic unstructured use.

[EP/CDE] Dietary nitrate is supported primarily for selected exercise-performance outcomes rather than classical muscle-damage recovery [100,101,102]. [EP] Creatine has established relevance to strength, lean mass, and repeated high-intensity performance; a conventional loading exposure is approximately 0.3 g/kg/day for 5–7 days followed by 3–5 g/day [103,104,105]. [CDE] Recovery-specific effects are heterogeneous and should not be inferred from performance evidence alone [104,106].

### 6.3. Collagen, Gelatin, and Connective-Tissue Support

[MR] Connective-tissue claims require a strict separation between surrogate and clinical outcomes. In eight healthy men, Shaw et al. [86] used a crossover design and found that 15 g vitamin C-enriched gelatin taken 1 h before intermittent loading increased circulating PINP by 153% at 4 h, compared with approximately 54–59% in placebo or 5 g conditions. PINP is a collagen-synthesis marker; this result does not demonstrate fewer injuries, faster rehabilitation, or earlier return to play.

**Figure 2:**
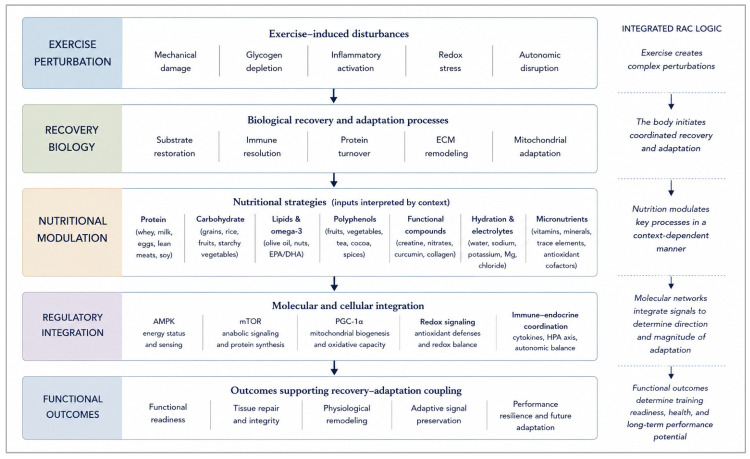
Mechanistic layers of exercise-recovery nutrition with claim-provenance interpretation. The vertical sequence is descriptive and does not establish a causal chain. Solid downward arrows represent broadly accepted process relations; mechanistic and RAC-derived elements remain MR or RH and require prospective validation. The right-hand column illustrates a conceptual integration hypothesis rather than a validated prediction. RAC = recovery–adaptation coupling; ECM = extracellular matrix.

[CDE/MR] A 15-participant pilot trial in resistance-trained men (seven collagen, eight placebo) reported maintenance of countermovement-jump height at 24 h in the collagen group, but collagen biomarkers did not change significantly, soreness increased in both groups, and maximal isometric force showed time effects without a clear comprehensive recovery advantage [88]. Therefore, collagen or gelatin plus vitamin C may be studied as an adjunct to targeted loading, but the cited evidence does not establish injury prevention, treatment efficacy, or return-to-play acceleration. Progressive loading, adequate energy and protein, and clinical rehabilitation remain primary (Figure 2).

Table 4 summarizes representative human samples, observed effects, precision and consistency limitations, and the boundary between applied evidence and mechanistic rationale.

## 7. Micronutrients, Hydration, and Deficiency-Driven Recovery

[EP] Micronutrients, hydration, and energy availability should be interpreted primarily through adequacy and deficiency correction rather than generic enhancement. Vitamin D, iron, calcium, and total energy availability require assessment in context; indiscriminate supplementation in already sufficient athletes is not justified [17,69,70,71,89,90,91,92,93,94,107,108,109,110,111].

[EP] Vitamin D supplementation is most defensible when baseline status is low and should be interpreted with season, sun exposure, geography, and prior supplementation [89,90,91]. Iron investigation should use ferritin together with hemoglobin, transferrin saturation, symptoms, and inflammatory context [107,108]. Suspected iron deficiency, RED-S, endocrine disturbance, recurrent illness, or bone-stress injury requires qualified medical and dietetic assessment. Magnesium supplementation should be tied to intake or clinical status rather than broad recovery claims [109,110].

[EP] Rehydration should account for measured fluid deficit, sodium loss, body-mass change, urine output, heat, travel, gastrointestinal tolerance, and the next session [69,70,71]. Recovery drinks are useful when they solve these constraints; they are not inherently superior to food. Hyponatremia risk, cardiovascular or renal disease, and other clinical considerations fall outside this framework and require professional oversight (Table 5).

## 8. Nutrient Timing, Chrononutrition, and Periodized Recovery

### 8.1. Immediate, Delayed, and Overnight Recovery Windows

[EP] Nutrient timing matters most when recovery time is short, appetite is impaired, or the next session is glycogen-dependent. For healthy adult athletes with substantial depletion and a 0–4 h recovery window, carbohydrate at approximately 1.0–1.2 g/kg/h, a high-quality protein feeding, and sodium-containing fluids can be prioritized [64,65,66,67,68,69,70,71,72,73,74,75,76,77,78,79,80,81,82,83,84,85]. When the next demanding session is more than a day away, total daily intake generally matters more than immediate timing. These ranges remain scenario-specific, not universal.

[CDE] After late competition, meal volume, fat and fiber load, caffeine exposure, and travel may impair sleep. Splitting recovery into an immediate digestible drink or snack followed by a later meal is a practical option, but direct performance evidence for a universal chrononutrition sequence is limited [18,95,96,97,112].

### 8.2. Competition Recovery Versus Adaptation-Oriented Training

[CDE] During tournaments or congested fixtures, incomplete glycogen restoration, dehydration, or force loss may justify aggressive carbohydrate and fluid-electrolyte restoration and selectively tested food-based polyphenol strategies [18,51,52,53,54,55,56,57,58,59,64,65,66,67,68,69,70,71]. [MR/RH] During adaptation-oriented blocks, avoiding chronic indiscriminate suppression of redox or inflammatory signals is biologically plausible, but the optimal intervention threshold is not established [10,11,41,42,43,44].

[RH] Training-fuel coupling provides adjacent conceptual background rather than validation of RAC [19]. The RAC-specific claim is that fuel, symptom control, and monitoring should be jointly matched to the intended stimulus and next demand; this combined decision rule remains to be tested prospectively.

### 8.3. Energy Availability, RED-S, and Systemic Recovery Capacity

[EP] Low energy availability can impair endocrine function, bone health, immune resilience, mood, sleep, protein turnover, and injury risk [17,92,93,94,111]. Persistent fatigue, recurrent illness or injury, menstrual or endocrine disturbance, declining performance, or unintended body-mass change should trigger qualified assessment of energy and carbohydrate availability before additional supplements are considered.

[EP] This issue is especially relevant in endurance, esthetic, weight-category, and high-load sports. Energy availability is a foundational condition for recovery; RAC does not diagnose or treat RED-S.

### 8.4. Training-Load Oscillation and Nutrient Periodization

[EP/CDE] Nutritional support should vary with training and competition demands rather than remain fixed. High-load days commonly require greater carbohydrate availability, distributed protein, fluid-electrolyte planning, and attention to sleep-linked feeding; lower-load days may require less aggressive symptom-control strategies while maintaining energy and micronutrient adequacy [3,20,21,22,23,24,25,26].

[RH] RAC hypothesizes that the intensity of recovery support should increase when a defined bottleneck threatens training continuity or competition readiness and decrease when recovery time allows adaptation. The threshold for this adjustment is not validated. Table 6 separates consensus practice, context-dependent evidence, mechanistic rationale, and RAC hypotheses.

## 9. Assessment of Recovery: From Subjective Symptoms to Multimodal Monitoring

### 9.1. Subjective, Functional, and Biochemical Measures

[EP] Subjective measures such as DOMS, fatigue, mood, sleep quality, appetite, and readiness are frequent and athlete-centered but are influenced by expectation and context. Functional tests such as countermovement jump, sprint output, strength, repeated-effort tasks, and sport-specific metrics are central because they are closer to applied readiness [20,21,22,23,24,25,26].

[EP/CDE] Biochemical markers such as creatine kinase, myoglobin, CRP, cytokines, oxidative-stress markers, iron status, and vitamin D may clarify persistent or unexplained fatigue. They rarely define recovery alone because timing, training history, muscle mass, hydration, assay variation, and individual response affect interpretation [27,28,29,30,31,37,98,99].

### 9.2. Wearables and Integrated Dashboards

[CDE] Wearables provide indirect information on sleep, heart-rate variability, resting heart rate, temperature, and load. Device algorithms, posture, timing, travel, alcohol, illness, heat, and sleep debt can alter values; a single reading should not classify readiness.

[RH] The revised RAC monitoring figure removes the green-amber-red classifier and all implied universal cut-offs. A research implementation should pre-specify a standardized within-athlete baseline window, use the same device and test conditions, estimate typical error or coefficient of variation, and use a validated sport- and device-specific smallest worthwhile change where available. Uncertain changes should be repeated, and interpretation should require persistent change or convergence with functional, subjective, and contextual information unless a medical red flag is present. These procedures are provisional research rules, not clinical decision thresholds.

**Figure 3:**
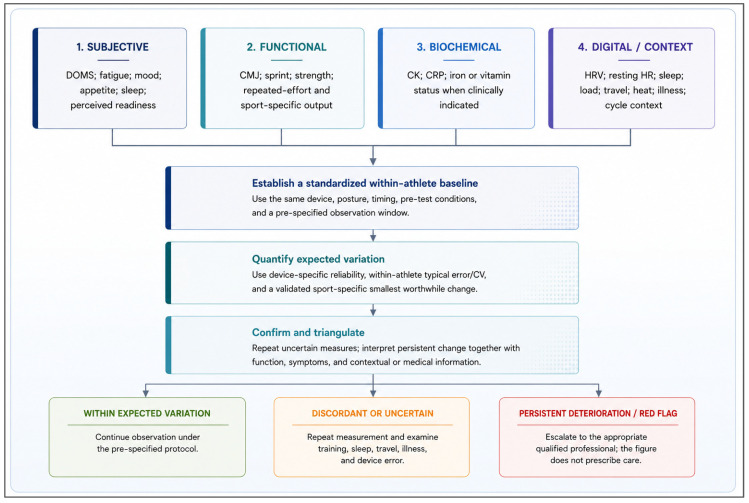
Multimodal recovery monitoring procedure. Subjective, functional, biochemical, and digital/contextual data are interpreted against standardized within-athlete baselines, measurement error, and validated smallest worthwhile changes where available. No universal cut-offs or device-independent classifications are proposed. Repeat measurement, cross-domain triangulation, and appropriate professional escalation are required. The procedure is a research-facing RAC hypothesis, not a diagnostic or treatment algorithm. CK = creatine kinase; CRP = C-reactive protein; HRV = heart-rate variability; CMJ = countermovement jump.

Figure 3 summarizes the proposed multimodal recovery monitoring procedure, integrating subjective, functional, biochemical, and digital/contextual information into a research-oriented interpretation framework.

Measurement uses and safeguards are summarized in Table 7.

## 10. Individual Variability and Candidate Recovery-Pattern Hypotheses

Athletes vary substantially in recovery kinetics. Training status, sex, age, body composition, sleep, menstrual-cycle symptoms, hormonal environment, energy availability, gut tolerance, injury history, baseline diet, psychological stress, and genetic factors may influence response. This variability justifies individualized observation, but it does not validate categorical “recovery phenotypes.”

[EP/CDE] Recovery studies frequently underrepresent women or inadequately characterize menstrual status, hormonal contraceptive use, energy availability, and cycle-related symptoms. Current evidence does not support rigid phase-based nutrition prescriptions for all female athletes. These variables should be recorded as context rather than used as deterministic categories [17,92,93,94,111,113,114,115].

[CDE] Selected small trials illustrate response heterogeneity: multi-week EPA/DHA altered strength loss or stiffness in some cohorts [48,49,50]; blueberry accelerated one isometric-strength outcome without reducing soreness [57]; and pomegranate findings arose from a nine-athlete protocol [58]. Such results support pre-specified subgroup analyses, not post hoc responder labels.

[MR] Dietary pattern, fiber diversity, fermented foods, polyphenol exposure, protein source, immune function, and gastrointestinal tolerance may influence response through the gut microbiome [116,117,118,119,120]. Current evidence is insufficient for routine microbiome-based recovery prescriptions.

[CDE] In extreme endurance exercise, genotype-associated differences in potassium, hematocrit, pH, and pCO_2_ have also been reported, suggesting that genetic context may contribute to inter-individual electrochemical and acid–base responses [121].

[RH] The previous applied “phenotype” classification has been withdrawn. Table 8 now presents candidate recovery patterns solely as prospective research hypotheses. A pattern must be defined before data collection, tested against a comparator, and validated with functional outcomes; it must not be used to diagnose or classify an individual athlete. Low energy availability, suspected iron deficiency, connective-tissue injury, endocrine disturbance, and persistent gastrointestinal symptoms are clinical assessment questions rather than RAC phenotypes.

## 11. Research-Facing Framework and Professional Boundaries

[RH] A research-facing RAC workflow begins by pre-specifying the next demand and the candidate recovery bottleneck. The purpose is to formulate a testable intervention hypothesis and outcome pattern, not to issue an automated recommendation. Established foundations—adequate energy, protein distribution, carbohydrate matched to workload, hydration, micronutrient sufficiency, and sleep—remain primary [3,4,5,6,17,18,20,21,22,23,24,25,26,64,65,66,67,68,69,70,71,72,73,74,75,76,77,78,79,80,81,82,83,84,85,86,87,88,89,90,91,92,93,94,95,96,97].

[RH] The candidate bottleneck must be operationalized with measurable inclusion criteria and a comparator before testing. Soreness, force loss, substrate depletion, dehydration, poor sleep, and low energy availability are not interchangeable, and improvement in one domain must not be assumed to represent tissue repair or readiness.

Professional boundaries are explicit. RAC does not diagnose or treat RED-S, iron deficiency, endocrine or menstrual disorders, gastrointestinal disease, injury, unexplained fatigue, or mental-health conditions. Return-to-play and rehabilitation decisions belong to the qualified medical and multidisciplinary team. Supplement use requires review of contraindications, medication interactions, renal or hepatic conditions, pregnancy, product contamination, and applicable anti-doping rules. Table 9 is therefore framed as a research matrix with safeguards rather than a clinical decision aid.

The previous decision tree was removed because its sequential presentation could imply validated prescriptive choices. Table 9 retains only the minimum research logic needed to define populations, comparators, outcomes, and professional safeguards.

Three worked applications are reported in Supplementary File S1 and are explicitly framed here as research examples and decision triggers rather than validated practice pathways.

## 12. Research Gaps and Future Directions

The recovery-nutrition literature is limited by small samples, untrained or recreationally active participants, heterogeneous products and doses, short follow-up, selective outcomes, sparse adverse-event reporting, and endpoints that do not map cleanly to sport performance. Trials are needed in trained populations, women, older athletes, team-sport environments, and real-world congested schedules; female-athlete studies should report menstrual status, hormonal contraceptive use, energy availability, and relevant symptoms [111,113,114,115,122,123,124].

Mechanistic research should move beyond isolated markers and test interacting networks involving AMPK, mTOR, PGC-1alpha, SIRT1, NAD+-related metabolism, inflammation resolution, redox signaling, collagen turnover, and immune-endocrine regulation [125,126,127,128]. Mechanistic findings should be paired with functional and longitudinal adaptation outcomes.

Future trials should pre-specify products, dose–response contrasts, timing, populations, adverse events, responder analyses, and clinically or sport-relevant outcomes. The most informative studies will test whether an intervention improves next-session function or long-term adaptation rather than only reducing soreness or a biomarker [18,106,112,129,130]. Table 10 summarizes these research priorities.

### 12.1. Testable Predictions of the Recovery–Adaptation Coupling Framework

To make RAC explicitly falsifiable, the broad predictions have been reformulated as study-ready hypotheses with defined populations, interventions, comparators, outcomes, and time horizons. Exact protocols should be registered prospectively and stratified by sex where sample size permits.

**P1**. In trained adult team-sport athletes completing two glycogen-demanding matches or sessions within 24 h, an aggressive recovery package initiated within 1 h (carbohydrate 1.0–1.2 g/kg/h for 4 h, approximately 0.3 g/kg protein, and individualized sodium/fluid replacement) will improve 24 h repeated-sprint performance and body-mass/fluid restoration versus an isoenergetic usual-practice recovery condition; gastrointestinal symptoms and adverse events will be recorded.
**P2**. In trained endurance athletes completing an 8- to 12-week adaptation block, daily high-dose antioxidant supplementation will improve selected acute symptom markers but produce smaller changes in pre-registered mitochondrial/redox-adaptation outcomes than a whole-food, energy-matched control, without superior end-block performance.
**P3**. In athletes prospectively stratified by a pre-defined and reproducible bottleneck (for example, documented glycogen limitation, persistent functional loss after damaging exercise, or sleep-linked next-day impairment), a bottleneck-matched intervention will improve the pre-specified functional recovery outcome over 4–6 weeks more than a standardized one-size-fits-all recovery package.
**P4**. In a prospective 8-week athlete cohort, a pre-registered model combining standardized subjective, functional, digital/contextual, and clinically indicated biochemical data will predict a pre-defined next-session performance decrement with better calibration and discrimination than soreness, creatine kinase, or heart-rate variability alone; performance will be evaluated in an external validation sample.
**P5**. In athletes undergoing a standardized 12-week tendon-rehabilitation program under clinical supervision, 15 g collagen or gelatin with vitamin C approximately 1 h before targeted loading will improve a validated tendon-specific functional score and progressive load tolerance more than matched loading with placebo or the same supplement provided at an unrelated time; adverse events and return-to-play criteria will be independently adjudicated.
**P6**. In trained athletes followed for 12–16 weeks, a periodized recovery-nutrition strategy that varies with pre-defined training density and adaptation goals will produce greater improvement in a pre-registered sport-performance outcome than a uniformly aggressive recovery strategy, without greater illness, injury, low-energy-availability indicators, or non-adherence.

These hypotheses can determine whether RAC adds predictive or intervention value beyond established recovery and sports-nutrition frameworks. Until such comparative studies are completed, RAC-specific rules remain RH.

### 12.2. Limitations of the Narrative Synthesis

Several limitations should be acknowledged. First, this is a narrative conceptual synthesis rather than a protocol-registered systematic review. No formal risk-of-bias scoring, certainty grading, PRISMA denominator, or pooled quantitative synthesis was performed, and the reconstructed search appendix cannot substitute for a contemporaneous search log. The framework can organize evidence and generate hypotheses but cannot estimate intervention-specific effect sizes or comparative certainty.

Second, the literature is heterogeneous in population, training status, sex-specific reporting, product formulation, dose, timing, comparator, adverse-event reporting, and outcome window. Many studies rely on small or untrained samples, short-term soreness outcomes, and isolated biomarkers. Claim-provenance labels improve transparency but do not correct these underlying limitations.

Third, RAC has not been validated as a predictive or decision algorithm. Its four lenses overlap, its monitoring procedure requires device- and sport-specific calibration, and its candidate recovery patterns are research hypotheses rather than classifications. Prospective studies must test whether RAC-guided designs improve prediction, readiness, tissue outcomes, or long-term adaptation beyond existing frameworks.

## 13. Conclusions

Recovery nutrition should be interpreted across the interval between exercise exposures rather than as the fastest possible suppression of soreness, inflammation, or oxidative stress. Existing evidence most consistently supports adequate energy availability, distributed high-quality protein, context-appropriate carbohydrate, and individualized fluid and sodium replacement. Other interventions require product-, population-, and outcome-specific interpretation.

RAC organizes this evidence around four overlapping lenses—damage attenuation, inflammation resolution, anabolic remodeling, and adaptive-signal preservation—and makes explicit the proposed links among next demand, bottleneck, adaptive consequence, and response verification. The framework does not establish that these links are causally independent or superior to existing recovery, sports-nutrition, fuel-periodization, or monitoring approaches.

At present, RAC should be restricted to evidence organization and hypothesis generation. It should not classify athletes, define universal monitoring thresholds, diagnose clinical conditions, prescribe supplements, or determine rehabilitation and return to play. Its value will depend on prospective comparative validation of the specific hypotheses and measurement procedures proposed here.

## Figures and Tables

**Table 1:** Comparison of RAC with adjacent recovery, sports-nutrition, fuel-periodization, and athlete-monitoring frameworks.

Framework/Sources	Core Focus and Principal Decision Rule	What is Not Explicitly Integrated	Relation to RAC
Recovery and performance consensus [3]	Balance stress and recovery across training, sleep, psychological, and physical modalities.	No nutrient-specific rule linking the next demand, adaptive cost, and response marker.	RAC retains the stress-recovery logic but adds a nutrition-specific bottleneck-to-marker sequence (RH).
Sports-nutrition positions and consensus [4,5,6,17,18]	Meet energy, macronutrient, fluid, micronutrient, and supplement-safety needs according to sport and workload.	No single framework jointly formalizes acute readiness, adaptive-signal preservation, and multimodal verification.	RAC does not replace these recommendations; EP foundations remain primary.
Fuel and carbohydrate periodization [10,11,19]	Match carbohydrate availability to the work required and the intended training stimulus.	Primarily fuel-centered; connective tissue, symptom-function discordance, and monitoring are outside its main scope.	RAC extends the context principle beyond carbohydrate, but this extension requires validation (RH).
Athlete-monitoring frameworks [20,21,22,23,24,25,26,27,28,29,30,31]	Interpret longitudinal subjective, functional, biochemical, and digital trends.	Monitoring identifies change but does not itself select a nutritional strategy or estimate adaptive trade-offs.	RAC proposes linking a nutritional hypothesis to a pre-specified monitoring pattern (RH).
Recovery–adaptation coupling (this review)	Jointly consider next demand, bottleneck, adaptive consequence, minimum sufficient intervention, and response verification.	No prospective evidence yet shows superiority over existing frameworks.	Contribution is conceptual integration and a falsifiable research agenda, not a validated algorithm.

Note. EP = established practice; RH = RAC hypothesis. The table compares organizing logic rather than grading the quality of entire frameworks. RAC is positioned as complementary to, not a replacement for, existing consensus guidance.

**Table 2:** Biological recovery processes and their nutritional targets.

Biological Process	Typical Signals/Markers	Recovery Implication	Nutritional Target
Mechanical disruption	Force loss, soreness, CK, myoglobin	Symptoms do not equal complete recovery	CDE/MR—Protein distribution, creatine, polyphenols, sufficient energy [1,2,32,33,34,35,36,37]
Inflammation	CRP, cytokines, leukocyte activity	Resolution is useful; chronic suppression may be problematic	CDE/MR—Omega-3 status, polyphenols, selective curcumin, whole-diet quality [38,39,40,46,47,48,49,50,51,52,53,54,55,56,57,58,59,60,61,62,63]
Redox signaling	ROS/RNS, antioxidant enzymes, oxidative damage markers	Signal preservation matters for adaptation	CDE/MR—Contextual antioxidant/polyphenol use, avoid chronic high-dose indiscriminate use [41,42,43,44,45]
Glycogen depletion	Repeated-session fatigue, substrate limitation	Performance may be impaired despite low soreness	EP—Carbohydrate timing, carbohydrate–protein co-ingestion, fluid/electrolytes [10,11,64,65,66,67,68,69,70,71]
Protein turnover	MPS/MPB balance, remodeling markers	Repair requires repeated anabolic opportunities	EP—High-quality protein, EAA/leucine, pre-sleep protein [72,73,74,75,76,77,78,79,80,81,82,83,84,85]
Connective-tissue remodeling	Tendon/ECM strain, pain/stiffness	Requires loading plus substrate support	MR/CDE—Collagen/gelatin + vitamin C, vitamin D sufficiency, energy adequacy [17,86,87,88,89,90,91,92,93,94]
Autonomic/sleep disruption	HRV, resting HR, sleep duration/quality	Systemic readiness may lag behind local tissue signals	CDE—Evening protein, carbohydrate where needed, hydration, caffeine management [20,21,22,23,24,25,26,69,70,71,95,96,97]

Note. CK = creatine kinase; CRP = C-reactive protein; ROS/RNS = reactive oxygen/nitrogen species; MPS/MPB = muscle protein synthesis/muscle protein breakdown; ECM = extracellular matrix; HRV = heart-rate variability. EP = established practice; CDE = context-dependent evidence; MR = mechanistic rationale. The status applies to the nutritional claim in each row, not to the biological process itself.

**Table 3:** Macronutrient and fluid strategies for recovery–adaptation coupling.

Strategy	Primary Mechanism	Best-Use Context	Main Caution
EP—High-quality protein distributed across the day	Repeated stimulation of MPS and remodeling	Resistance training, high-damage sessions, older or anabolic-resistant athletes	Population, total daily intake, energy availability, age, and clinical status determine application [72,73,74,75,76,77,78,79,80,81,82]
EP—Leucine/EAA-rich feeding	Anabolic signaling and substrate availability	Low appetite, rapid recovery meals, plant-protein planning	Protein quality and total energy remain relevant [75,77,78,79,80,81,82]
CDE—Pre-sleep protein	Overnight amino acid availability	Late sessions, hypertrophy blocks, long fasting interval	Should not replace daytime protein distribution [83,84]
EP—Carbohydrate restoration	Glycogen resynthesis and repeated-session capacity	Two-a-day training, tournaments, endurance and team sports	The 1.0–1.2 g/kg/h range is relevant mainly after substantial depletion with <8 h to the next demanding session [64,65,66,67,68]
EP/CDE—Carbohydrate–protein co-ingestion	Practical mixed recovery and energy support	Short windows, poor appetite, travel	Not always superior when carbohydrate intake is already sufficient [64,65,66,67,68]
CDE—Omega-3 fatty acids	Membrane and inflammation-resolution environment	Longer-term recovery support, inflammation-prone profiles	Effects are not immediate and dose/context matter [46,47,48,49,50]
EP—Fluid and sodium	Plasma volume and thermoregulatory recovery	Sweaty sessions, heat, travel, next-day competition	Use measured loss and clinical context; plain water alone may be inadequate after heavy sweating [69,70,71]

Note. MPS = muscle protein synthesis; EAA = essential amino acids. EP = established practice; CDE = context-dependent evidence. Status labels identify claim provenance, not formal certainty. Quantitative ranges apply primarily to healthy adult athletic populations and must be adjusted for body size, sex, age, training mode, total intake, clinical status, and recovery interval.

**Table 4:** Representative human evidence for bioactive compounds and functional foods: samples, outcomes, limitations, and claim boundaries.

Intervention and Cited Sample	Representative Exposure	Observed Outcomes and Limitations	Claim Status and Boundary
Tart cherry: *n* = 14 male crossover [53]; *n* = 16 semi-professional male players [56]	12 fl oz blend twice daily for 8 d or 30 mL concentrate twice daily for 8 d	Strength loss averaged 22% vs. 4% in one crossover trial, but tenderness and elbow angle were null; selected recovery outcomes improved in another small trial. Products and outcomes differ.	CDE—possible use in defined damaging or congested scenarios; no universal dose or class effect.
Pomegranate: *n* = 9 elite weightlifters [58]	High-volume juice exposure around two weightlifting sessions	Selected performance, soreness, CK/LDH, and CRP differences; very small crossover sample and unusually high-volume protocol limit generalization.	CDE—exploratory and product-specific.
Blueberry: *n* = 10 physically active women [57]	Five 200 g smoothie servings before and through 36 h after eccentric exercise	Treatment × time interaction for peak isometric torque (*p* = 0.047); soreness was not reduced and other strength patterns were less clear.	CDE—small crossover signal requiring replication.
Curcumin: *n* = 20 randomized pilot [60]; *n* = 17 male crossover [61]	200 mg twice daily phytosome or 2.5 g twice daily around eccentric exercise	Selected pain and biomarker effects; one study reported VAS changes ~1.0–1.9 cm, CK −22% to −29%, and jump +15%, with wide 90% limits. Formulation and dose differ greatly.	CDE—short-term research exposure only; chronic safety and optimal formulation unresolved.
Dietary nitrate/beetroot [100,101,102]	Acute pre-exercise protocols	Evidence cited here primarily concerns oxygen cost and performance, not post-damage recovery; no recovery-specific dose is validated.	EP for selected performance uses; not established for EIMD recovery.
Creatine [103,104,105,106]	Loading ~0.3 g/kg/day for 5–7 d, then 3–5 g/day	Established strength and power relevance; recovery findings are mixed and heterogeneous.	EP for strength/power; CDE for recovery-specific claims.
Gelatin/collagen + vitamin C: *n* = 8 mechanistic crossover [86]; *n* = 15 pilot [88]	15 g ~1 h before loading; or 15 g/day for 7 d before damaging exercise	PINP increased 153% in the mechanistic study; pilot functional findings were selective and collagen markers were null. No injury-prevention or return-to-play endpoint was demonstrated.	MR/CDE—adjunct research hypothesis; not a treatment or return-to-play recommendation.

Note. EP = established practice; CDE = context-dependent evidence; MR = mechanistic rationale; EIMD = exercise-induced muscle damage. These are claim-provenance labels, not GRADE ratings. Sample sizes and representative effects are shown to prevent isolated positive protocols from being interpreted as universal recommendations. Adverse-event reporting was limited in the small recovery trials; absence of reported serious events does not establish long-term safety.

**Table 5:** Micronutrients, hydration, and energy availability in recovery physiology.

Nutrient/System	Recovery Relevance	Best-Use Logic	Assessment Cue
EP—Vitamin D	Muscle function, bone/connective tissue, immune context	Correct deficiency; maintain sufficiency in low-sun seasons [89,90,91]	25(OH)D status, injury history, geography
EP—Iron	Oxygen transport, fatigue, endurance recovery	Correct documented deficiency under medical/dietetic supervision [107,108]	Ferritin, hemoglobin, transferrin saturation, inflammation context
CDE—Magnesium	Neuromuscular function and energy metabolism	Prioritize dietary adequacy; supplement if intake/status suggests need [109,110]	Dietary intake, cramps/fatigue context, clinical status
EP—Calcium	Bone, neuromuscular function, low-energy-availability contexts	Ensure adequacy in athletes with RED-S or bone-risk profiles [17,92,93,94,111]	Dietary intake, bone stress history
EP—Zinc/selenium	Immune-redox support	Correct low intake; avoid excess [5,6,17]	Diet history, illness frequency, clinical judgment
EP—Sodium/electrolytes	Fluid retention and sweat-loss replacement	Use after heavy sweating, heat, travel, short recovery windows [69,70,71]	Body-mass change, urine color/specific gravity, sweat rate
EP—Total energy availability	Systemic capacity for repair and adaptation	Assess and restore energy availability; do not layer supplements over persistent under-fueling [17,92,93,94,111]	Body mass trend, menstrual/endocrine signs, mood, fatigue, injury risk

Note. 25(OH)D = 25-hydroxyvitamin D; RED-S = relative energy deficiency in sport. EP = established practice; CDE = context-dependent evidence. Suspected deficiency, RED-S, endocrine or menstrual disturbance, recurrent illness, bone-stress injury, or unexplained fatigue requires appropriate medical and dietetic assessment.

**Table 6:** Nutrient timing and periodization scenarios with claim-provenance labels.

Timing Scenario	Primary Goal	Priority Strategy	Adaptation Caution
0–4 h between sessions	Rapid substrate and fluid restoration	EP—Carbohydrate 1.0–1.2 g/kg/h initially; protein 0.25–0.40 g/kg; sodium/fluid [64,65,66,67,68,69,70,71,72,73,74,75,76,77,78,79,80,81,82,83,84,85]	Low concern if performance urgency is high
Same-day evening after late competition	Recovery without sleep disruption	CDE—Split digestible carbohydrate/protein feeding, sodium/fluid, and manage caffeine [18,64,65,66,67,68,69,70,71,95,96,97,112]	Avoid overfeeding or heavy foods that impair sleep
Overnight recovery	Extend anabolic and systemic recovery	CDE—Approximately 30–40 g pre-sleep protein, hydration, regular sleep schedule [83,84,95,96,97]	Keep timing practical and athlete-specific
High-DOMS microcycle	Limit excessive force loss and soreness	CDE—Protein, selective polyphenols/curcumin, creatine, carbohydrate, and sleep [46,47,48,49,50,51,52,53,54,55,56,57,58,59,60,61,62,63,95,96,97,98,99,100,101,102,103,104,105,106]	Do not use soreness alone as recovery endpoint
Adaptation-oriented endurance block	Support remodeling while preserving signaling	CDE/MR—Periodized carbohydrate, adequate protein, whole-food antioxidants [10,11,41,42,43,44]	Avoid chronic high-dose antioxidant suppression
Tournament/congested fixtures	Short-term readiness	CDE—Aggressive recovery feeding, fluids/electrolytes, tested polyphenol-rich foods [18,51,52,53,54,55,56,57,58,59,64,65,66,67,68,69,70,71]	Adaptation concerns are secondary to performance continuity
Return-to-play tissue remodeling	Load tolerance and tissue repair	MR—Energy adequacy, protein, collagen/gelatin + vitamin C with loading [17,86,87,88,89,90,91,92,93,94]	Do not confuse pain reduction with tissue capacity

Note. EP = established practice; CDE = context-dependent evidence; MR = mechanistic rationale. Timing urgency increases when the recovery window is short and the next session is demanding. Quantitative ranges apply primarily to healthy adult athletes and require adjustment for population, total intake, gastrointestinal tolerance, and clinical status.

**Table 7:** Recovery-monitoring domains, uses, and measurement safeguards.

Domain	Examples	Appropriate Use	Required Measurement Safeguard
EP—Subjective	DOMS, fatigue, mood, readiness, appetite, sleep quality	Frequent athlete-centered trend information	Use stable wording and timing; interpret expectation and contextual effects.
EP—Functional	CMJ, sprint, strength, repeated-effort, sport-specific output	Direct assessment of performance capacity	Standardize protocol; establish reliability and meaningful change for the test.
EP/CDE—Biochemical	CK, CRP, myoglobin, cytokines, redox or micronutrient markers	Targeted evaluation of deficiency, persistent fatigue, or systemic stress	Use clinical indication, standardized sampling, reference context, and qualified interpretation.
CDE—Digital	HRV, resting HR, sleep, temperature, load metrics	Longitudinal pattern detection	Account for device, algorithm, posture, timing, travel, illness, and technical error.
EP—Contextual	Training load, travel, heat, illness, menstrual-cycle symptoms, nutrition history	Explains why other signals may change	Requires accurate athlete-practitioner communication and privacy safeguards.
RH—Integrated RAC procedure	Pre-specified baseline, typical error/CV, smallest worthwhile change, repeat confirmation	Research comparison of multimodal versus single-marker prediction	No universal threshold; prospective validation and calibration are required before action rules.

Note. CMJ = countermovement jump; CK = creatine kinase; CRP = C-reactive protein; HRV = heart-rate variability; CV = coefficient of variation; EP = established practice; CDE = context-dependent evidence; RH = RAC hypothesis. The table specifies measurement safeguards and does not define diagnostic or return-to-play thresholds.

**Table 8:** Candidate recovery-pattern hypotheses and prospective validation requirements.

Candidate Pattern (All RH)	Pre-Specified Study Definition	Candidate Comparison	Validation Outcome and Boundary
High symptom plus force-loss pattern	Elevated standardized DOMS plus a reproducible decline in CMJ, sprint, or strength after a defined damaging session	Context-matched recovery strategy vs. standardized recovery	Rate of functional recovery; symptoms alone are insufficient.
Slow functional-recovery pattern	Functional output remains beyond the test’s typical error at a pre-specified time point	Targeted protein/energy/creatine strategy vs. control	Functional trajectory and training completion; requires test reliability.
Glycogen-limited pattern	Repeated-session performance decline after verified glycogen-demanding exercise and inadequate carbohydrate exposure	Rapid carbohydrate restoration vs. usual intake	Next-session output and tolerance; not inferred from fatigue alone.
Sleep-sensitive pattern	Reproducible association between late-session context, standardized sleep measures, and next-day function	Evening meal/caffeine protocol vs. habitual practice	Sleep plus next-day function; wearable values alone are insufficient.
Low-energy-availability presentation	Pre-specified screening indicates possible low energy availability or RED-S risk	Qualified multidisciplinary assessment, not an RAC supplement trial	Medical/dietetic outcomes; RAC does not diagnose or treat RED-S.
Connective-tissue risk presentation	Clinically characterized tendon/ligament problem with a standardized loading program	Adjunct timed collagen/gelatin study vs. matched loading control	Pain, validated function, load tolerance, and return-to-play outcomes under clinical oversight.
GI-tolerance constraint	Reproducible GI symptoms that limit planned recovery intake	Tolerability-adapted foods/timing vs. standard plan	Energy intake, symptoms, adherence; persistent symptoms require clinical assessment.

Note. RH = RAC hypothesis; DOMS = delayed-onset muscle soreness; CMJ = countermovement jump; GI = gastrointestinal; RED-S = relative energy deficiency in sport. These are prospective research patterns, not diagnostic labels or currently validated classifications.

**Table 9:** Research-facing RAC matrix with professional safeguards.

Research Scenario	Evidence-Supported Foundation	RAC-Specific Research Question	Required Safeguards and Outcomes
Congested competition/short recovery	EP—carbohydrate, protein, individualized sodium/fluid, sleep opportunity	Does a context-matched package improve next-session function beyond usual evidence-based recovery?	Pre-specify depletion, timing, GI tolerance, body-mass recovery, and sport-specific output.
High-damage exercise block	EP/CDE—adequate protein/energy; CDE—selected creatine or polyphenol strategies	Does targeting documented force loss outperform a standardized package?	Use function plus symptoms; monitor product safety and avoid equating lower soreness with repair.
Adaptation-oriented block	EP—adequate energy/protein; CDE—carbohydrate periodization	Does less aggressive symptom control preserve adaptation without reducing training quality?	Longitudinal performance and adaptation outcomes; monitor under-fueling and illness.
Connective-tissue rehabilitation	EP—progressive loading and clinical rehabilitation; MR/CDE—collagen/gelatin adjunct	Does timing an adjunct before loading improve validated function or load tolerance?	Medical oversight; no supplement-led return-to-play decision; record adverse events.
Sleep or autonomic strain	EP/CDE—sleep opportunity, caffeine management, tolerable evening intake	Can a standardized evening protocol improve sleep and next-day function?	Device reliability, travel/illness context, repeat measures, and functional outcomes.
Possible low energy availability or micronutrient deficiency	EP—qualified assessment and correction of documented insufficiency	No RAC algorithm should be tested before the underlying clinical question is addressed.	Medical/dietetic evaluation; ferritin/hemoglobin and other tests only when indicated; supplements secondary.

Note. EP = established practice; CDE = context-dependent evidence; MR = mechanistic rationale; The matrix is a research-planning aid, not a diagnostic, treatment, supplementation, or return-to-play tool. Medical, dietetic, anti-doping, and product-safety requirements take precedence.

**Table 10:** Research priorities for precision recovery nutrition.

Research Gap	Rationale	Recommended Design Feature	Priority Outcome
Athlete-specific evidence	Trained athletes respond differently from untrained participants	Trials in trained, elite, female, and team-sport samples	Function plus recovery kinetics
Dose–response uncertainty	Many interventions vary by dose and product composition	Standardized products and multiple dosing arms	Dose-specific benefit-risk profile
Timing and periodization	Same strategy may differ between competition and adaptation blocks	Compare acute, chronic, and periodized protocols	Readiness and adaptation markers
Responder phenotypes	Group means hide individual recovery patterns	Preplanned responder analyses and baseline profiling	Personalized response prediction
Integrated monitoring	Single markers are insufficient	Subjective, functional, biochemical, and wearable dashboards	Decision accuracy and sport outcomes
Long-term adaptation risk	Acute symptom control may alter training signals	Longitudinal training studies	Performance remodeling, not only DOMS
Real-world implementation	Athletes face travel, limited appetite, and schedule constraints	Pragmatic trials in teams and competitions	Adherence, tolerance, and next-session performance

Note. All entries are research priorities (RH), not current treatment recommendations. Priority outcomes emphasize next-session readiness, functional recovery kinetics, long-term adaptation, implementation feasibility, safety, and sport-specific performance [18,20,21,22,23,24,25,26,27,28,29,30,31,98,99,106,112,122,123,124,125,126,127,128,129,130].

## Data Availability

No new data were created or analyzed in this study. Data sharing is not applicable to this article.

## Supplementary Materials

The following supporting information can be downloaded at: https://www.mdpi.com/article/10.3390/nu18152523/s1, Supplementary File S1: Applied RAC case examples and decision triggers; Supplementary File S2: Reconstructed search strategy, narrative selection pathway, operational criteria, claim-provenance rules, and complete 130-record evidence inventory.

## Abbreviations

RAC	Recovery–adaptation coupling
DOMS	Delayed-onset muscle soreness
EIMD	Exercise-induced muscle damage
CK	Creatine kinase
CRP	C-reactive protein
ROS	Reactive oxygen species
RNS	Reactive nitrogen species
MPS	Muscle protein synthesis
MPB	Muscle protein breakdown
EAA	Essential amino acids
AMPK	AMP-activated protein kinase
mTOR	Mechanistic target of rapamycin
PGC-1alpha	Peroxisome proliferator-activated receptor gamma coactivator 1-alpha
NAD+	Nicotinamide adenine dinucleotide
RED-S	Relative energy deficiency in sport
HRV	Heart-rate variability
CMJ	Countermovement jump
RPE	Rating of perceived exertion
EP	Established practice
CDE	Context-dependent evidence
MR	Mechanistic rationale
RH	Recovery–adaptation coupling hypothesis
