# Supplementary material for: Nutritional Strategies for Recovery–Adaptation Coupling After Exercise: From Muscle Damage to Performance Remodeling
Source: Nutrients. 2026 Aug 4;18(15):2523. doi: 10.3390/nu18152523 (PMC13468400; doi:10.3390/nu18152523)
Supplement: Supplementary file 1 [file nutrients-18-02523-s001.zip › nutrients-4470369-File S1.pdf]

nutrients

MDPI

Supplementary Material

Supplementary File S1: Applied RAC Case Examples and Decision Triggers

The following examples illustrate the intended use of RAC as a heuristic workflow. They are not validated universal algorithms or substitutes for sport-specific clinical and performance judgment.

Example 1: congested team-sport schedule. After a late match with high sweating, repeated accelerations, and a demanding session or fixture within 24 h, the next-demand question prioritizes readiness over adaptation. The RAC response emphasizes carbohydrate plus high-quality protein, sodium-containing fluids guided by body-mass change and urine output, familiar portable foods, and selective food-based polyphenols only when previous use or the literature supports improved force recovery or soreness control. Escalation is justified when morning readiness, sleep, HRV/resting heart rate, and CMJ, sprint, or repeated-effort performance deteriorate together relative to the athlete's baseline; if functional markers remain suppressed, nutrition should be paired with load adjustment rather than simply adding more supplements.

Example 2: adaptation-oriented endurance or mixed training block. When the next session is not immediately performance-critical and the goal is mitochondrial, metabolic, or aerobic remodeling, RAC prioritizes adequate energy availability, protein distribution, and planned carbohydrate rather than routine high-dose antioxidant or anti-inflammatory supplementation. Whole-food polyphenols remain compatible with a high-quality diet, but concentrated products are reserved for unusually damaging sessions or competition phases. Escalation is triggered less by soreness alone than by persistent fatigue, declining repeated-session output, poor sleep, low appetite, or signs of low energy availability.

Example 3: connective-tissue or return-to-play context. When tendon stiffness, recurrent pain, or load intolerance is the dominant bottleneck, the nutritional priority shifts from rapid symptom suppression to tissue-capacity support. Collagen or gelatin with vitamin C may be timed before targeted loading, but the decision remains dependent on progressive mechanical stimulus, energy sufficiency, protein adequacy, vitamin D or iron status when indicated, and longitudinal pain-stiffness-load-tolerance patterns. Reduced pain is not interpreted as complete tissue recovery unless accompanied by improved functional capacity and progressive load tolerance.

Across all examples, the sequence is the same: choose the smallest effective intervention, predefine the expected marker pattern, and reassess after repeated exposures. Practical triggers should be individualized because meaningful change differs across sports, tests, devices, and athletes.
