# Supplementary material for: Nutritional Strategies for Recovery–Adaptation Coupling After Exercise: From Muscle Damage to Performance Remodeling
Source: Nutrients. 2026 Aug 4;18(15):2523. doi: 10.3390/nu18152523 (PMC13468400; doi:10.3390/nu18152523)
Supplement: Supplementary file 1 [file nutrients-18-02523-s001.zip › nutrients-4470369-supplementary file 2.pdf]

Supplementary File S2: Reconstructed search strategy, narrative selection pathway, operational criteria, claim-provenance rules, and complete 130-record evidence inventory.

Purpose and status. This file improves transparency for the narrative review "Nutritional Strategies for Recovery-Adaptation Coupling after Exercise: From Muscle Damage to Performance Remodeling." It provides database-ready reconstructed search syntax, explicit eligibility and prioritization rules, the narrative selection pathway, claim-provenance definitions, and an inventory of every record cited in the revised manuscript. It is a reporting and audit supplement, not a protocol-registered systematic-review appendix.

Transparency limitation. The original searches were iterative and citation-led. Contemporaneous day-level execution logs, complete result counts, duplicate counts, exclusion counts, and database exports were not retained. The syntax below was reconstructed on 27 July 2026 from the concepts, sources, and boundaries documented in the manuscript; it must not be represented as the original archived search log. No retrospective PRISMA denominator has been fabricated.

S2.1. Databases, coverage, fields, and filters

Coverage extended from database inception through 31 May 2026. PubMed/MEDLINE, Scopus, and Web of Science Core Collection were the principal databases. Google Scholar was used only for citation chaining, title verification, "cited by" exploration, and backward/forward screening of key reviews, consensus statements, and position stands. Eligible sources were peer-reviewed articles in English. Conference abstracts, editorials without substantive evidence, non-peer-reviewed material, duplicate publications, and records lacking sufficient methodological or outcome detail were de-prioritized. No human-only filter was imposed because mechanistic cell, animal, engineered-tissue, and clinical physiology papers were used selectively to explain pathways, but never as stand-alone support for sport-performance recommendations.

The database-specific strings in Section S2.2 reproduce five concept blocks: recovery biology; macronutrients and hydration; bioactive compounds and supplements; connective tissue, micronutrients, and energy availability; and monitoring, individual variability, and implementation. Search fields and filters are stated beneath each database heading.

S2.2. Reconstructed database-ready search syntax

The strings below are intentionally broad because the synthesis combined intervention evidence, accepted practice, mechanistic explanation, and monitoring literature. Where a database limits query length, each block can be run separately and combined during citation screening. The end-date restriction is 31 May 2026.

S2.2.1. PubMed/MEDLINE

Fields: Title/Abstract ([tiab]) for concept terms. Filters: English language; publication date from database inception through 31 May 2026. No publication-type or human-only filter was applied.

```
Block 1 – recovery biology and nutritional modulation

(("exercise-induced muscle damage" OR EIMD OR "delayed-onset muscle soreness" OR DOMS OR "muscle recovery" OR "exercise recovery" OR "inflammation resolution" OR cytokine* OR "oxidative stress" OR "redox signaling" OR "reactive oxygen species" OR "reactive nitrogen species")[Title/Abstract] AND (nutrition* OR diet* OR supplement* OR protein OR carbohydrate* OR creatine OR polyphenol* OR curcumin OR "omega-3" OR nitrate* OR collagen OR gelatin OR micronutrient* OR hydration OR electrolyte*)[Title/Abstract] AND (athlete* OR trained OR exercise OR "resistance exercise" OR endurance OR "team sport*" OR eccentric OR "repeated sprint*" OR competition)[Title/Abstract]) AND English[lang] AND ("1900/01/01"[Date - Publication] : "2026/05/31"[Date - Publication])

Block 2 – macronutrients, glycogen, hydration, and timing

(("muscle protein synthesis" OR protein OR leucine OR "essential amino acid*" OR EAA OR casein OR "pre-sleep protein" OR glycogen OR carbohydrate* OR "carbohydrate availability" OR "glycogen resynthesis" OR hydration OR rehydration OR sodium OR electrolyte* OR "fluid replacement" OR chrononutrition OR "nutrient timing")[Title/Abstract] AND (recovery OR adaptation OR readiness OR fatigue OR
```

```
performance)[Title/Abstract] AND (athlete* OR trained OR exercise OR sport*)[Title/Abstract]) AND English[lang] AND ("1900/01/01"[Date - Publication] : "2026/05/31"[Date - Publication])
```

Block 3 — bioactive compounds and selected supplements

```
((polyphenol* OR "tart cherry" OR Montmorency OR pomegranate OR blueberry OR berries OR curcumin OR turmeric OR creatine OR nitrate* OR beetroot OR "omega-3" OR EPA OR DHA)[Title/Abstract] AND (recovery OR DOMS OR soreness OR "muscle damage" OR inflammation OR strength OR power OR "repeated sprint*" OR performance OR adaptation)[Title/Abstract] AND (athlete* OR trained OR exercise OR sport*)[Title/Abstract]) AND English[lang] AND ("1900/01/01"[Date - Publication] : "2026/05/31"[Date - Publication])
```

Block 4 — connective tissue, micronutrients, and systemic recovery

```
((collagen OR gelatin OR tendon OR ligament OR "extracellular matrix" OR ECM OR "vitamin C" OR "vitamin D" OR iron OR ferritin OR magnesium OR calcium OR zinc OR selenium OR "energy availability" OR "relative energy deficiency in sport" OR RED-S OR REDs)[Title/Abstract] AND (recovery OR rehabilitation OR "return to play" OR remodeling OR adaptation OR fatigue OR injury OR performance)[Title/Abstract] AND (athlete* OR trained OR exercise OR sport*)[Title/Abstract]) AND English[lang] AND ("1900/01/01"[Date - Publication] : "2026/05/31"[Date - Publication])
```

Block 5 — monitoring, variability, sex-specific context, and implementation

```
(("heart rate variability" OR HRV OR wearable* OR "training load" OR readiness OR "countermovement jump" OR CMJ OR biomarker* OR "creatine kinase" OR sleep OR menstrual OR "hormonal contraceptive*" OR female OR microbiome OR "gut tolerance" OR "gastrointestinal tolerance" OR phenotype* OR responder*)[Title/Abstract] AND (recovery OR adaptation OR fatigue OR performance OR monitoring)[Title/Abstract] AND (athlete* OR trained OR exercise OR sport*)[Title/Abstract]) AND English[lang] AND ("1900/01/01"[Date - Publication] : "2026/05/31"[Date - Publication])
```

S2.2.2. Scopus

Fields: title, abstract, and author keywords (TITLE-ABS-KEY). Filters: publication year before 2027; English; journal source; article or review document type.

Block 1 — recovery biology and nutritional modulation

```
TITLE-ABS-KEY(("exercise-induced muscle damage" OR EIMD OR "delayed-onset muscle soreness" OR DOMS OR "muscle recovery" OR "exercise recovery" OR "inflammation resolution" OR cytokine* OR "oxidative stress" OR "redox signaling" OR "reactive oxygen species" OR "reactive nitrogen species") AND (nutrition* OR diet* OR supplement* OR protein OR carbohydrate* OR creatine OR polyphenol* OR curcumin OR "omega-3" OR nitrate* OR collagen OR gelatin OR micronutrient* OR hydration OR electrolyte*) AND (athlete* OR trained OR exercise OR "resistance exercise" OR endurance OR "team sport*" OR eccentric OR "repeated sprint*" OR competition)) AND PUBYEAR < 2027 AND LIMIT-TO(LANGUAGE, "English") AND LIMIT-TO(SRCTYPE, "j") AND (LIMIT-TO(DOCTYPE, "ar") OR LIMIT-TO(DOCTYPE, "re"))
```

Block 2 — macronutrients, glycogen, hydration, and timing

```
TITLE-ABS-KEY(("muscle protein synthesis" OR protein OR leucine OR "essential amino acid*" OR EAA OR casein OR "pre-sleep protein" OR glycogen OR carbohydrate* OR "carbohydrate availability" OR "glycogen resynthesis" OR hydration OR rehydration OR sodium OR electrolyte* OR "fluid replacement" OR chrononutrition OR "nutrient timing") AND (recovery OR adaptation OR readiness OR fatigue OR performance) AND (athlete* OR trained OR exercise OR sport*)) AND PUBYEAR < 2027 AND LIMIT-TO(LANGUAGE, "English") AND LIMIT-TO(SRCTYPE, "j") AND (LIMIT-TO(DOCTYPE, "ar") OR LIMIT-TO(DOCTYPE, "re"))
```

Block 3 — bioactive compounds and selected supplements

```
TITLE-ABS-KEY((polyphenol* OR "tart cherry" OR Montmorency OR pomegranate OR blueberry OR berries OR curcumin OR turmeric OR creatine OR nitrate* OR beetroot OR "omega-3" OR EPA OR DHA) AND (recovery OR DOMS OR soreness OR "muscle damage" OR inflammation OR strength OR power OR "repeated sprint*" OR performance OR adaptation) AND (athlete* OR trained OR exercise OR sport*)) AND PUBYEAR < 2027 AND LIMIT-TO(LANGUAGE, "English") AND LIMIT-TO(SRCTYPE, "j") AND (LIMIT-TO(DOCTYPE, "ar") OR LIMIT-TO(DOCTYPE, "re"))
```

Block 4 — connective tissue, micronutrients, and systemic recovery

```
TITLE-ABS-KEY((collagen OR gelatin OR tendon OR ligament OR "extracellular matrix" OR ECM OR "vitamin C" OR "vitamin D" OR iron OR ferritin OR magnesium OR calcium OR zinc OR selenium OR "energy availability" OR "relative energy deficiency in sport" OR RED-S OR REDs) AND (recovery OR rehabilitation OR "return to play" OR remodeling OR adaptation OR fatigue OR injury OR performance) AND (athlete* OR trained OR exercise OR sport*)) AND PUBYEAR < 2027 AND LIMIT-TO(LANGUAGE, "English") AND LIMIT-TO(SRCTYPE, "j") AND (LIMIT-TO(DOCTYPE, "ar") OR LIMIT-TO(DOCTYPE, "re"))
```

Block 5 — monitoring, variability, sex-specific context, and implementation

```
TITLE-ABS-KEY(("heart rate variability" OR HRV OR wearable* OR "training load" OR readiness OR "countermovement jump" OR CMJ OR biomarker* OR "creatine kinase" OR sleep OR menstrual OR "hormonal contraceptive*" OR female OR microbiome OR "gut tolerance" OR "gastrointestinal tolerance" OR phenotype* OR responder*) AND (recovery OR adaptation OR fatigue OR performance OR monitoring) AND (athlete* OR trained OR exercise OR sport*)) AND PUBYEAR < 2027 AND LIMIT-TO(LANGUAGE, "English") AND LIMIT-TO(SRCTYPE, "j") AND (LIMIT-TO(DOCTYPE, "ar") OR LIMIT-TO(DOCTYPE, "re"))
```

S2.2.3. Web of Science Core Collection

Fields: Topic (TS: title, abstract, author keywords, and Keywords Plus). Filters: English; Article or Review; publication years 1900–2026. Records published after 31 May 2026 were not eligible for the manuscript cutoff.

Block 1 — recovery biology and nutritional modulation

```
TS=(("exercise-induced muscle damage" OR EIMD OR "delayed-onset muscle soreness" OR DOMS OR "muscle recovery" OR "exercise recovery" OR "inflammation resolution" OR cytokine* OR "oxidative stress" OR "redox signaling" OR "reactive oxygen species" OR "reactive nitrogen species") AND (nutrition* OR diet*
```

```
OR supplement* OR protein OR carbohydrate* OR creatine OR polyphenol* OR curcumin OR "omega-3" OR nitrate* OR collagen OR gelatin OR micronutrient* OR hydration OR electrolyte*) AND (athlete* OR trained OR exercise OR "resistance exercise" OR endurance OR "team sport*" OR eccentric OR "repeated sprint*" OR competition)) AND LA=(English) AND DT=(Article OR Review) AND PY=(1900-2026)
```

Block 2 — macronutrients, glycogen, hydration, and timing

```
TS=(("muscle protein synthesis" OR protein OR leucine OR "essential amino acid*" OR EAA OR casein OR "pre-sleep protein" OR glycogen OR carbohydrate* OR "carbohydrate availability" OR "glycogen resynthesis" OR hydration OR rehydration OR sodium OR electrolyte* OR "fluid replacement" OR chrononutrition OR "nutrient timing") AND (recovery OR adaptation OR readiness OR fatigue OR performance) AND (athlete* OR trained OR exercise OR sport*)) AND LA=(English) AND DT=(Article OR Review) AND PY=(1900-2026)
```

Block 3 — bioactive compounds and selected supplements

```
TS=((polyphenol* OR "tart cherry" OR Montmorency OR pomegranate OR blueberry OR berries OR curcumin OR turmeric OR creatine OR nitrate* OR beetroot OR "omega-3" OR EPA OR DHA) AND (recovery OR DOMS OR soreness OR "muscle damage" OR inflammation OR strength OR power OR "repeated sprint*" OR performance OR adaptation) AND (athlete* OR trained OR exercise OR sport*)) AND LA=(English) AND DT=(Article OR Review) AND PY=(1900-2026)
```

Block 4 — connective tissue, micronutrients, and systemic recovery

```
TS=((collagen OR gelatin OR tendon OR ligament OR "extracellular matrix" OR ECM OR "vitamin C" OR "vitamin D" OR iron OR ferritin OR magnesium OR calcium OR zinc OR selenium OR "energy availability" OR "relative energy deficiency in sport" OR RED-S OR REDs) AND (recovery OR rehabilitation OR "return to play" OR remodeling OR adaptation OR fatigue OR injury OR performance) AND (athlete* OR trained OR exercise OR sport*)) AND LA=(English) AND DT=(Article OR Review) AND PY=(1900-2026)
```

Block 5 — monitoring, variability, sex-specific context, and implementation

```
TS=(("heart rate variability" OR HRV OR wearable* OR "training load" OR readiness OR "countermovement jump" OR CMJ OR biomarker* OR "creatine kinase" OR sleep OR menstrual OR "hormonal contraceptive*" OR female OR microbiome OR "gut tolerance" OR "gastrointestinal tolerance" OR phenotype* OR responder*) AND (recovery OR adaptation OR fatigue OR performance OR monitoring) AND (athlete* OR trained OR exercise OR sport*)) AND LA=(English) AND DT=(Article OR Review) AND PY=(1900-2026)
```

S2.2.4. Google Scholar citation tracking

Google Scholar was not used to construct a countable denominator. Searches were phrase-based and followed by "Cited by," "Related articles," and backward-reference screening. Representative complete phrases were: "exercise-induced muscle damage" nutrition recovery athlete; "recovery nutrition" athlete muscle damage; "carbohydrate periodization" recovery adaptation; polyphenol exercise recovery muscle soreness; curcumin DOMS randomized; collagen gelatin vitamin C tendon exercise; athlete monitoring recovery heart-rate variability; and female athlete recovery menstrual cycle. Key consensus statements, position stands, systematic reviews, and influential trials identified in the database searches were also used as citation-chaining seeds.

S2.3. Operational eligibility, prioritization, and de-prioritization criteria

Evidence was evaluated at claim level rather than assigning one global label to an entire article. A record could contribute to more than one outcome domain. The following operational rules were used in the revised synthesis and in the post hoc audit of the final cited corpus.

Criterion	Operational definition
Relevance	At least one of four domains: recovery biology; nutritional strategy; applied recovery/performance outcome; monitoring/interpretation variable.
Direct transferability	Trained or athletic participants, or an exercise model closely matching the proposed use. Evidence from untrained or clinical populations required an explicit transfer rationale.
Adequate comparator	Concurrent placebo, usual-practice, matched-diet, or within-participant condition exposed to the same exercise and measurement schedule.
Product characterization	Formulation, dose, timing, and duration stated clearly enough to interpret the exposure.
Functional alignment	Outcome relevant to the proposed application and recovery window overlapping the next athletic demand.
Exploratory sample threshold	Fewer than 10 participants per arm treated as exploratory unless supported by larger, replicated, or higher-level evidence.
Mechanistic-only use	Cell, animal, engineered-tissue, clinical-physiology, or surrogate-marker evidence used to explain pathways, not as stand-alone support for performance, prevention, rehabilitation, or return-to-play claims.
De-prioritization	Poorly characterized intervention; no suitable comparator; non-specific wellness endpoint; isolated biomarker without functional interpretation; mismatched recovery window; conclusion exceeding design.

Criterion	Operational definition
Context modifiers	Baseline nutritional status, total energy intake, training status, sex-specific reporting, adverse-event reporting, gastrointestinal tolerance, and proximity to the next demand.
Conflicting findings	Greater interpretive weight to athlete-relevant samples, characterized products, adequate comparators, functional outcomes, and matched time windows; null/inconsistent findings retained when they narrowed the best-use context.
Selection personnel and disagreement	The original narrative process was not conducted by a fixed duplicate-screening team. No prospective disagreement log or inter-rater statistic exists. The revised inventory is an author-level audit of cited records, not independent duplicate screening.

S2.4. Narrative evidence-selection pathway and transparency limit

Supplementary Figure S1 depicts the actual logic of the narrative process: targeted database and citation searches; relevance screening; design and transferability prioritization; de-prioritization of low-transferability or biomarker-only claims; claim-level mapping to outcome domain and provenance status; and audit of the final 130 cited records. Because contemporaneous search exports and counts were not retained, the figure intentionally contains no retrospective PRISMA-style numbers.

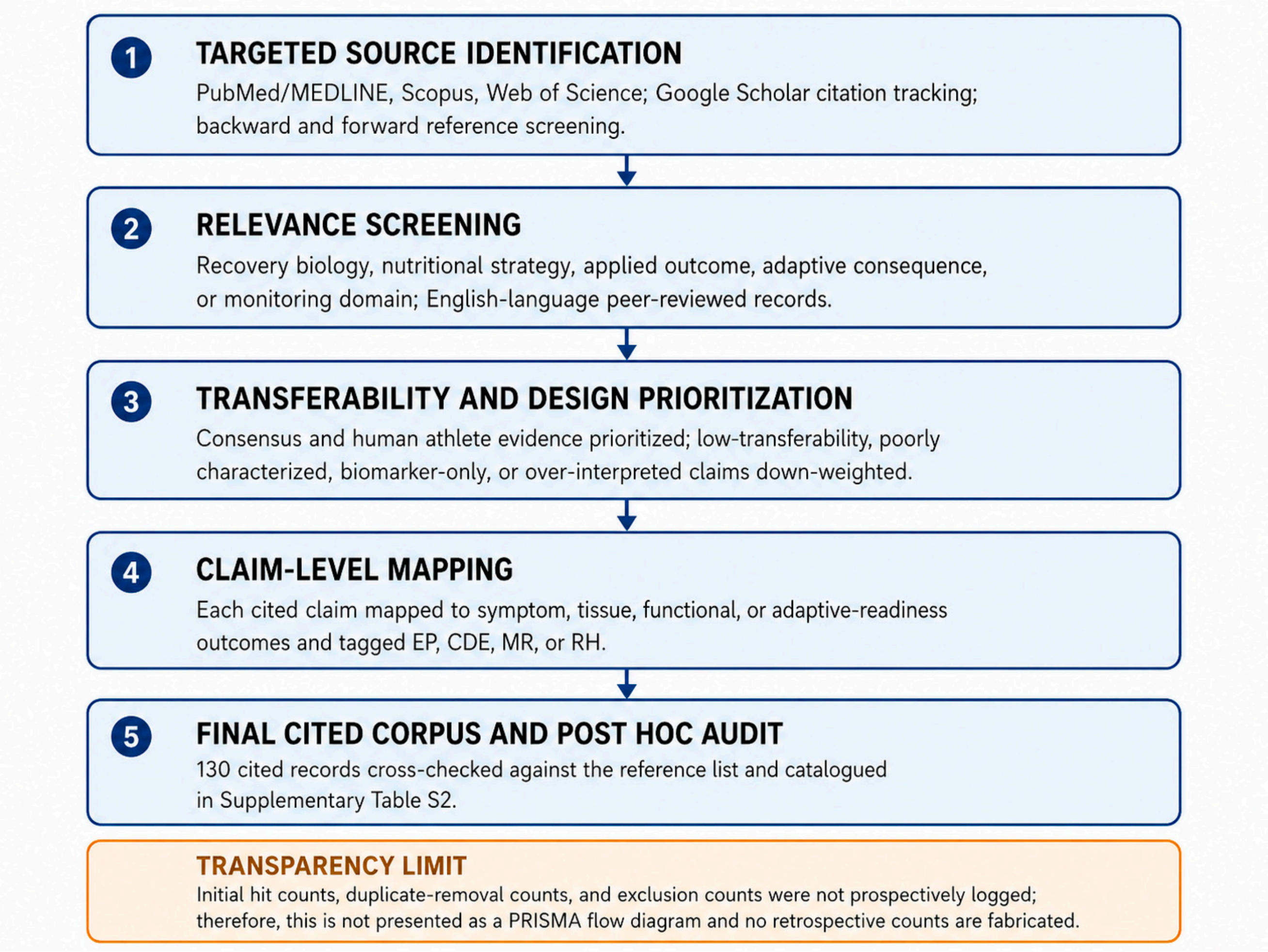

Supplementary Figure S1. Narrative evidence-selection and citation-audit pathway. The final audit includes all 130 cited records, but no claim is made that the pathway reconstructs missing search counts or independent duplicate screening.

S2.5. Outcome-domain and claim-provenance rules

Outcome domains are non-exclusive. Symptom recovery includes DOMS, fatigue, mood, appetite, sleep quality, and perceived readiness. Tissue recovery includes structural, muscle-damage, inflammatory, redox, collagen-turnover, hydration, and micronutrient-status markers. Functional recovery includes force, power, sprint, repeated-effort, endurance, and sport-specific performance. Adaptive readiness includes chronic remodeling, signaling, and training-response outcomes relevant to future capacity.

Claim-provenance labels identify the source and maturity of a statement; they are not GRADE ratings, formal certainty grades, or numerical rankings. EP denotes established practice supported by consensus/position guidance and replicated human evidence in the relevant setting. CDE denotes context-dependent human evidence that is heterogeneous, product-specific, population-limited, or inconsistent. MR denotes mechanistic rationale or surrogate evidence without demonstrated applied benefit. RH denotes an author-generated RAC linkage, category, threshold, or prediction that has not been prospectively validated.

Label	Rule	Representative example
EP — established practice	Consensus/position guidance plus replicated human evidence in a relevant population.	Adequate energy availability; distributed high-quality protein; carbohydrate restoration in a short recovery window; individualized fluid/sodium replacement.
CDE — context-dependent evidence	Human evidence exists but varies by product, dose, population, comparator, outcome, or time window.	Polyphenol products, curcumin, omega-3 status, recovery-specific creatine effects, pre-sleep protein outside the best-studied populations.
MR — mechanistic rationale	Biological plausibility or surrogate outcome without validated applied benefit.	Gelatin/collagen effects on collagen-synthesis markers; redox and molecular pathway explanations.
RH — RAC hypothesis	Author-generated linkage, classification, threshold, monitoring rule, or prediction not prospectively validated.	Recovery-pattern categories, bottleneck matching, multimodal monitoring procedure, adaptive-signal trade-off rules, P1–P6 predictions.

S2.6. Complete 130-record evidence inventory

Supplementary Table S1 lists every record cited in the revised manuscript. Record type, primary synthesis domain, and principal claim role were assigned descriptively for transparency. These entries are not formal study-level risk-of-bias judgments or certainty grades. An article may support multiple claims; the table records its principal use in this review.

Supplementary Table S1. Complete inventory of records cited in the revised RAC narrative synthesis.

Ref.	Full citation	Record type	Primary synthesis domain	Principal role / claim status
1	Peake, J.M.; Neubauer, O.; Della Gatta, P.A.; Nosaka, K. Muscle damage and inflammation during recovery from exercise. J. Appl. Physiol. 2017, 122, 559-570. https://doi.org/10.1152/japplphysiol.00971.2016	Narrative review	Recovery, fueling, and adaptation foundations	EP/MR foundation for recovery biology, adaptive signaling, and conceptual boundaries.
2	Owens, D.J.; Twist, C.; Cobley, J.N.; Howatson, G.; Close, G.L. Exercise-induced muscle damage: What is it, what causes it and what are the nutritional solutions? Eur. J. Sport Sci. 2019, 19, 71-85. https://doi.org/10.1080/17461391.2018.1505957	Narrative review	Recovery, fueling, and adaptation foundations	EP/MR foundation for recovery biology, adaptive signaling, and conceptual boundaries.
3	Kellmann, M.; Bertollo, M.; Bosquet, L.; Brink, M.; Coutts, A.J.; Duffield, R.; Erlacher, D.; Halson, S.L.; Hecksteden, A.; Heidari, J.; et al. Recovery and performance in sport: Consensus statement. Int. J. Sports Physiol. Perform. 2018, 13, 240-245. https://doi.org/10.1123/ijspp.2017-0759	Consensus statement	Recovery, fueling, and adaptation foundations	EP or EP/CDE foundation; retained for accepted practice, safety, or methodological context.
4	Thomas, D.T.; Erdman, K.A.; Burke, L.M. Position of the Academy of Nutrition and Dietetics, Dietitians of Canada, and the American College of Sports Medicine: Nutrition and athletic performance. J. Acad. Nutr. Diet. 2016, 116, 501-528. https://doi.org/10.1016/j.jand.2015.12.006	Professional position statement	Recovery, fueling, and adaptation foundations	EP or EP/CDE foundation; retained for accepted practice, safety, or methodological context.
5	Kerksick, C.M.; Wilborn, C.D.; Roberts, M.D.; Smith-Ryan, A.; Kleiner, S.M.; Jager, R.; Collins, R.; Cooke, M.; Davis, J.N.; Galvan, E.; et al. ISSN exercise and sports nutrition review update: Research and recommendations. J. Int. Soc. Sports Nutr. 2018, 15, 38. https://doi.org/10.1186/s12970-018-0242-y	Narrative review/position synthesis	Recovery, fueling, and adaptation foundations	EP or EP/CDE foundation; retained for accepted practice, safety, or methodological context.
6	Maughan, R.J.; Burke, L.M.; Dvorak, J.; Larson-Meyer, D.E.; Peeling, P.; Phillips, S.M.; Rawson, E.S.; Walsh, N.P.; Garthe, I.; Geyer, H.; et al. IOC consensus statement: Dietary supplements and the high-performance athlete. Br. J. Sports Med. 2018, 52, 439-455. https://doi.org/10.1136/bjsports-2018-099027	Consensus statement	Recovery, fueling, and adaptation foundations	EP or EP/CDE foundation; retained for accepted practice, safety, or methodological context.
7	Egan, B.; Zierath, J.R. Exercise metabolism and the molecular regulation of skeletal muscle adaptation. Cell Metab. 2013, 17, 162-184. https://doi.org/10.1016/j.cmet.2012.12.012	Mechanistic review	Recovery, fueling, and adaptation foundations	EP/MR foundation for recovery biology, adaptive signaling, and conceptual boundaries.
8	Hawley, J.A.; Hargreaves, M.; Joyner, M.J.; Zierath, J.R. Integrative biology of exercise. Cell 2014, 159, 738-749. https://doi.org/10.1016/j.cell.2014.10.029	Integrative review	Recovery, fueling, and adaptation foundations	EP/MR foundation for recovery biology, adaptive signaling, and conceptual boundaries.
9	Powers, S.K.; Jackson, M.J. Exercise-induced oxidative stress: Cellular mechanisms and impact on muscle force production. Physiol. Rev. 2008, 88, 1243-1276. https://doi.org/10.1152/physrev.00031.2007	Mechanistic review	Recovery, fueling, and adaptation foundations	EP/MR foundation for recovery biology, adaptive signaling, and conceptual boundaries.
10	Impey, S.G.; Hearris, M.A.; Hammond, K.M.; Bartlett, J.D.; Louis, J.; Close, G.L.; Morton, J.P. Fuel for the work required: A theoretical framework for carbohydrate periodization and the glycogen threshold hypothesis. Sports Med. 2018, 48, 1031-1048. https://doi.org/10.1007/s40279-018-0867-7	Theoretical framework	Recovery, fueling, and adaptation foundations	EP/MR foundation for recovery biology, adaptive signaling, and conceptual boundaries.
11	Bartlett, J.D.; Hawley, J.A.; Morton, J.P. Carbohydrate availability and exercise training adaptation: Too much of a good thing? Eur. J. Sport Sci. 2015, 15, 3-12. https://doi.org/10.1080/17461391.2014.920926	Conceptual review	Recovery, fueling, and adaptation foundations	EP/MR foundation for recovery biology, adaptive signaling, and conceptual boundaries.
12	Mănescu, D.C.; Tudor, A.; Mănescu, A.M.; Mărgărit, I.R.; Mănescu, C.O.; Prisăcaru, C.; Păun, L.; Tudor, V. Antioxidants and Exercise: A Redox-Informed Framework for Training Adaptation, Performance, and Recovery. Antioxidants 2026, 15, 456. https://doi.org/10.3390/antiox15040456	Author-group conceptual review	Conceptual positioning and adjacent frameworks	RH positioning only; not used as empirical validation or intervention-efficacy evidence.
13	Mănescu, D.C.; Plăstoi, C.D.; Petre, R.L.; Mărgărit, I.R.; Mănescu, A.M.; Pîrvan, A. Metabolic Overdrive in Elite Sport: A Systems Model of AMPK-mTOR Oscillation, NAD+ Economy, and Epigenetic Drift. Int. J. Mol. Sci. 2026, 27, 1817. https://doi.org/10.3390/ijms27041817	Author-group conceptual framework	Conceptual positioning and adjacent frameworks	RH positioning only; not used as empirical validation or intervention-efficacy evidence.
14	Mănescu, A.M.; Hangu, S.Ș.; Mănescu, D.C. Nutritional Supplements for Muscle Hypertrophy: Mechanisms and Morphology-Focused Evidence. Nutrients 2025, 17, 3603. https://doi.org/10.3390/nu17223603	Author-group narrative review	Conceptual positioning and adjacent frameworks	RH positioning only; not used as empirical validation or intervention-efficacy evidence.
15	Stoian, M.; Mănescu, D.C. Training-Fuel Coupling (TFC): A Molecular Sports Nutrition Framework for Energy Availability, Chrono-Nutrition, and Performance Optimization. Nutrients 2026, 18, 693. https://doi.org/10.3390/nu18040693	Author-group conceptual framework	Conceptual positioning and adjacent frameworks	RH positioning only; not used as empirical validation or intervention-efficacy evidence.
16	Mănescu, D.C. Training Load Oscillation and Epigenetic Plasticity: Molecular Pathways Connecting Energy Metabolism and Athletic Personality. Int. J. Mol. Sci. 2026, 27, 792. https://doi.org/10.3390/ijms27020792	Author-group conceptual paper	Conceptual positioning and adjacent frameworks	RH positioning only; not used as empirical validation or intervention-efficacy evidence.
17	MacInnis, M.J.; Gibala, M.J. Physiological adaptations to interval training and the role of exercise intensity. J. Physiol. 2017, 595, 2915-2930. https://doi.org/10.1113/JP273196	Narrative review	Conceptual positioning and adjacent frameworks	EP/MR foundation for recovery biology, adaptive signaling, and conceptual boundaries.
18	Proske, U.; Morgan, D.L. Muscle damage from eccentric exercise: Mechanism, mechanical signs, adaptation and clinical applications. J. Physiol. 2001, 537, 333-345. https://doi.org/10.1111/j.1469-7793.2001.00333.x	Mechanistic review	EIMD, inflammation, redox, and adaptation biology	EP/MR foundation for recovery biology, adaptive signaling, and conceptual boundaries.
19	Clarkson, P.M.; Hubal, M.J. Exercise-induced muscle damage in humans. Am. J. Phys. Med. Rehabil. 2002, 81, S52-S69. https://doi.org/10.1097/00002060-200211001-00007	Narrative review	EIMD, inflammation, redox, and adaptation biology	EP/MR foundation for recovery biology, adaptive signaling, and conceptual boundaries.
20	Hyldahl, R.D.; Hubal, M.J. Lengthening our perspective: Morphological, cellular, and molecular responses to eccentric exercise. Muscle Nerve 2014, 49, 155-170. https://doi.org/10.1002/mus.24077	Mechanistic review	EIMD, inflammation, redox, and adaptation biology	EP/MR foundation for recovery biology, adaptive signaling, and conceptual boundaries.

Ref.	Full citation	Record type	Primary synthesis domain	Principal role / claim status
21	Tidball, J.G. Inflammatory processes in muscle injury and repair. Am. J. Physiol. Regul. Integr. Comp. Physiol. 2005, 288, R345-R353. https://doi.org/10.1152/ajpregu.00454.2004	Mechanistic review	EIMD, inflammation, redox, and adaptation biology	EP/MR foundation for recovery biology, adaptive signaling, and conceptual boundaries.
22	Tidball, J.G. Regulation of muscle growth and regeneration by the immune system. Nat. Rev. Immunol. 2017, 17, 165-178. https://doi.org/10.1038/nri.2016.150	Mechanistic review	EIMD, inflammation, redox, and adaptation biology	EP/MR foundation for recovery biology, adaptive signaling, and conceptual boundaries.
23	Özdemir, K.; Demir, Y. Biochemical markers of muscle damage and recovery: Insights from exercise physiology and molecular biology. Tissue Cell 2026, 101, 103406. https://doi.org/10.1016/j.tice.2026.103406	Narrative biomarker review	EIMD, inflammation, redox, and adaptation biology	EP/MR foundation for recovery biology, adaptive signaling, and conceptual boundaries.
24	Serhan, C.N.; Savill, J. Resolution of inflammation: The beginning programs the end. Nat. Immunol. 2005, 6, 1191-1197. https://doi.org/10.1038/ni1276	Mechanistic review	EIMD, inflammation, redox, and adaptation biology	EP/MR foundation for recovery biology, adaptive signaling, and conceptual boundaries.
25	Serhan, C.N. Pro-resolving lipid mediators are leads for resolution physiology. Nature 2014, 510, 92-101. https://doi.org/10.1038/nature13479	Mechanistic review	EIMD, inflammation, redox, and adaptation biology	EP/MR foundation for recovery biology, adaptive signaling, and conceptual boundaries.
26	Powers, S.K.; Duarte, J.; Kavazis, A.N.; Talbert, E.E. Reactive oxygen species are signalling molecules for skeletal muscle adaptation. Exp. Physiol. 2010, 95, 1-9. https://doi.org/10.1113/expphysiol.2009.050526	Mechanistic review	EIMD, inflammation, redox, and adaptation biology	EP/MR foundation for recovery biology, adaptive signaling, and conceptual boundaries.
27	Gomez-Cabrera, M.C.; Domenech, E.; Romagnoli, M.; Arduini, A.; Borras, C.; Pallardo, F.V.; Sastre, J.; Vina, J. Oral administration of vitamin C decreases muscle mitochondrial biogenesis and hampers training-induced adaptations in endurance performance. Am. J. Clin. Nutr. 2008, 87, 142-149. https://doi.org/10.1093/ajcn/87.1.142	Human intervention study	EIMD, inflammation, redox, and adaptation biology	CDE/MR; supports caution about chronic indiscriminate antioxidant suppression.
28	Ristow, M.; Zarse, K.; Oberbach, A.; Kloting, N.; Birringer, M.; Kiehntopf, M.; Stumvoll, M.; Kahn, C.R.; Bluher, M. Antioxidants prevent health-promoting effects of physical exercise in humans. Proc. Natl. Acad. Sci. USA 2009, 106, 8665-8670. https://doi.org/10.1073/pnas.0903485106	Human intervention study	EIMD, inflammation, redox, and adaptation biology	CDE/MR; supports caution about chronic indiscriminate antioxidant suppression.
29	Paulsen, G.; Cumming, K.T.; Holden, G.; Hallen, J.; Ronnestad, B.R.; Sveen, O.; Skaug, A.; Paur, I.; Bastani, N.E.; Ostgaard, H.N.; et al. Vitamin C and E supplementation hampers cellular adaptation to endurance training in humans. J. Physiol. 2014, 592, 1887-1901. https://doi.org/10.1113/jphysiol.2013.267419	Human intervention study	EIMD, inflammation, redox, and adaptation biology	CDE/MR; supports caution about chronic indiscriminate antioxidant suppression.
30	Merry, T.L.; Ristow, M. Do antioxidant supplements interfere with skeletal muscle adaptation to exercise training? J. Physiol. 2016, 594, 5135-5147. https://doi.org/10.1113/JP270654	Narrative review	EIMD, inflammation, redox, and adaptation biology	CDE/MR; supports caution about chronic indiscriminate antioxidant suppression.
31	Schoenfeld, B.J. The mechanisms of muscle hypertrophy and their application to resistance training. J. Strength Cond. Res. 2010, 24, 2857-2872. https://doi.org/10.1519/JSC.0b013e3181e840f3	Mechanistic review	EIMD, inflammation, redox, and adaptation biology	EP/MR foundation for recovery biology, adaptive signaling, and conceptual boundaries.
32	Smith, G.I.; Atherton, P.; Reeds, D.N.; Mohammed, B.S.; Rankin, D.; Rennie, M.J.; Mittendorfer, B. Omega-3 polyunsaturated fatty acids augment the muscle protein anabolic response to hyperinsulinaemia-hyperaminoacidaemia in healthy young and middle-aged men and women. Clin. Sci. 2011, 121, 267-278. https://doi.org/10.1042/CS20100597	Human metabolic intervention	Omega-3 status and recovery	CDE; product-, dose-, population-, and time-dependent omega-3 evidence.
33	Smith, G.I.; Atherton, P.; Reeds, D.N.; Mohammed, B.S.; Rankin, D.; Rennie, M.J.; Mittendorfer, B. Dietary omega-3 fatty acid supplementation increases the rate of muscle protein synthesis in older adults: A randomized controlled trial. Am. J. Clin. Nutr. 2011, 93, 402-412. https://doi.org/10.3945/ajcn.110.005611	Randomized human intervention	Omega-3 status and recovery	CDE; product-, dose-, population-, and time-dependent omega-3 evidence.
34	Tsuchiya, Y.; Yanagimoto, K.; Nakazato, K.; Hayamizu, K.; Ochi, E. Eicosapentaenoic and docosahexaenoic acids-rich fish oil supplementation attenuates strength loss and limited joint range of motion after eccentric contractions: A randomized, double-blind, placebo-controlled, parallel-group trial. Eur. J. Appl. Physiol. 2016, 116, 1179-1188. https://doi.org/10.1007/s00421-016-3373-3	Randomized controlled trial	Omega-3 status and recovery	CDE; product-, dose-, population-, and time-dependent omega-3 evidence.
35	Ochi, E.; Tsuchiya, Y.; Yanagimoto, K. Effect of eicosapentaenoic acids-rich fish oil supplementation on motor nerve function after eccentric contractions. J. Int. Soc. Sports Nutr. 2017, 14, 23. https://doi.org/10.1186/s12970-017-0176-9	Human intervention study	Omega-3 status and recovery	CDE; product-, dose-, population-, and time-dependent omega-3 evidence.
36	Tsuchiya, Y.; Yanagimoto, K.; Ueda, H.; Ochi, E. Supplementation of eicosapentaenoic acid-rich fish oil attenuates muscle stiffness after eccentric contractions of human elbow flexors. J. Int. Soc. Sports Nutr. 2019, 16, 19. https://doi.org/10.1186/s12970-019-0283-x	Human intervention study	Omega-3 status and recovery	CDE; product-, dose-, population-, and time-dependent omega-3 evidence.
37	Bowtell, J.L.; Kelly, V. Fruit-derived polyphenol supplementation for athlete recovery and performance. Sports Med. 2019, 49, 3-23. https://doi.org/10.1007/s40279-018-0998-x	Narrative review	Polyphenols, fruit products, and curcumin	CDE; selected small human trials and synthesis, not a universal class recommendation.
38	Howatson, G.; McHugh, M.P.; Hill, J.A.; Brouner, J.; Jewell, A.P.; van Someren, K.A.; Shave, R.E.; Howatson, S.A. Influence of tart cherry juice on indices of recovery following marathon running. Scand. J. Med. Sci. Sports 2010, 20, 843-852. https://doi.org/10.1111/j.1600-0838.2009.01005.x	Human intervention study	Polyphenols, fruit products, and curcumin	CDE; selected small human trials and synthesis, not a universal class recommendation.
39	Connolly, D.A.J.; McHugh, M.P.; Padilla-Zakour, O.I.; Carlson, L.; Sayers, S.P. Efficacy of a tart cherry juice blend in preventing the symptoms of muscle damage. Br. J. Sports Med. 2006, 40, 679-683. https://doi.org/10.1136/bjsm.2005.025429	Randomized crossover trial	Polyphenols, fruit products, and curcumin	CDE; selected small human trials and synthesis, not a universal class recommendation.
40	Bowtell, J.L.; Sumners, D.P.; Dyer, A.; Fox, P.; Mileva, K.N. Montmorency cherry juice reduces muscle damage caused by intensive strength exercise. Med. Sci. Sports Exerc. 2011, 43, 1544-1551. https://doi.org/10.1249/MSS.0b013e31820e5adc	Randomized controlled trial	Polyphenols, fruit products, and curcumin	CDE; selected small human trials and synthesis, not a universal class recommendation.
41	Bell, P.G.; Walshe, I.H.; Davison, G.W.; Stevenson, E.; Howatson, G. Montmorency cherries reduce the oxidative stress and inflammatory responses to repeated days high-intensity stochastic cycling. Nutrients 2014, 6, 829-843. https://doi.org/10.3390/nu6020829	Randomized controlled trial	Polyphenols, fruit products, and curcumin	CDE; selected small human trials and synthesis, not a universal class recommendation.
42	Bell, P.G.; Stevenson, E.; Davison, G.W.; Howatson, G. The effects of Montmorency tart cherry concentrate supplementation on recovery following prolonged, intermittent exercise. Nutrients 2016, 8, 441. https://doi.org/10.3390/nu8070441	Randomized controlled trial	Polyphenols, fruit products, and curcumin	CDE; selected small human trials and synthesis, not a universal class recommendation.

Ref.	Full citation	Record type	Primary synthesis domain	Principal role / claim status
43	McLeay, Y.; Barnes, M.J.; Mundel, T.; Hurst, S.M.; Hurst, R.D.; Stannard, S.R. Effect of New Zealand blueberry consumption on recovery from eccentric exercise-induced muscle damage. J. Int. Soc. Sports Nutr. 2012, 9, 19. https://doi.org/10.1186/1550-2783-9-19	Small crossover trial	Polyphenols, fruit products, and curcumin	CDE; selected small human trials and synthesis, not a universal class recommendation.
44	Ammar, A.; Turki, M.; Chtourou, H.; Hammouda, O.; Trabelsi, K.; Kallel, C.; Abdelkarim, O.; Hoekelmann, A.; Souissi, N.; Bailey, S.J. Pomegranate supplementation accelerates recovery of muscle damage and soreness and inflammatory markers after a weightlifting training session. PLoS ONE 2016, 11, e0160305. https://doi.org/10.1371/journal.pone.0160305	Small crossover trial	Polyphenols, fruit products, and curcumin	CDE; selected small human trials and synthesis, not a universal class recommendation.
45	Torregrosa-Garcia, A.; Avila-Gandia, V.; Luque-Rubia, A.J.; Abellan-Ruiz, M.S.; Querol-Calderon, M.; Lopez-Roman, F.J. Pomegranate extract improves maximal performance of trained cyclists after an exhausting endurance trial: A randomized controlled trial. Nutrients 2019, 11, 721. https://doi.org/10.3390/nu11040721	Randomized controlled trial	Polyphenols, fruit products, and curcumin	CDE; selected small human trials and synthesis, not a universal class recommendation.
46	Drobnic, F.; Riera, J.; Appendino, G.; Togni, S.; Franceschi, F.; Valle, X.; Pons, A.; Tur, J. Reduction of delayed onset muscle soreness by a novel curcumin delivery system (Meriva): A randomised, placebo-controlled trial. J. Int. Soc. Sports Nutr. 2014, 11, 31. https://doi.org/10.1186/1550-2783-11-31	Randomized controlled pilot trial	Polyphenols, fruit products, and curcumin	CDE; selected small human trials and synthesis, not a universal class recommendation.
47	Nicol, L.M.; Rowlands, D.S.; Fazakerly, R.; Kellett, J. Curcumin supplementation likely attenuates delayed onset muscle soreness (DOMS). Eur. J. Appl. Physiol. 2015, 115, 1769-1777. https://doi.org/10.1007/s00421-015-3152-6	Small crossover trial	Polyphenols, fruit products, and curcumin	CDE; selected small human trials and synthesis, not a universal class recommendation.
48	McFarlin, B.K.; Venable, A.S.; Henning, A.L.; Sampson, J.N.; Pennel, K.; Vingren, J.L.; Hill, D.W. Reduced inflammatory and muscle damage biomarkers following oral supplementation with bioavailable curcumin. BBA Clin. 2016, 5, 72-78. https://doi.org/10.1016/j.bbacli.2016.02.003	Human intervention study	Polyphenols, fruit products, and curcumin	CDE; selected small human trials and synthesis, not a universal class recommendation.
49	Beba, M.; Djordjevic, B.; Vujovic, A.; Stojanovic, E.; Scanlan, A.T.; Milanovic, Z. The effect of curcumin supplementation on delayed-onset muscle soreness, inflammation, and muscle damage: A systematic review and meta-analysis. Phytother. Res. 2022, 36, 3331-3345. https://doi.org/10.1002/ptr.7477	Systematic review and meta-analysis	Polyphenols, fruit products, and curcumin	CDE; selected small human trials and synthesis, not a universal class recommendation.
50	Jentjens, R.; Jeukendrup, A.E. Determinants of post-exercise glycogen synthesis during short-term recovery. Sports Med. 2003, 33, 117-144. https://doi.org/10.2165/00007256-200333020-00004	Narrative review	Carbohydrate, glycogen, fluid, and sodium	EP or EP/CDE foundation; retained for accepted practice, safety, or methodological context.
51	Burke, L.M.; Hawley, J.A.; Wong, S.H.S.; Jeukendrup, A.E. Carbohydrates for training and competition. J. Sports Sci. 2011, 29, S17-S27. https://doi.org/10.1080/02640414.2011.585473	Narrative review/position guidance	Carbohydrate, glycogen, fluid, and sodium	EP or EP/CDE foundation; retained for accepted practice, safety, or methodological context.
52	Ivy, J.L.; Katz, A.L.; Cutler, C.L.; Sherman, W.M.; Coyle, E.F. Muscle glycogen synthesis after exercise: Effect of time of carbohydrate ingestion. J. Appl. Physiol. 1988, 64, 1480-1485. https://doi.org/10.1152/jappl.1988.64.4.1480	Human experimental study	Carbohydrate, glycogen, fluid, and sodium	EP/CDE; supports short-window glycogen restoration under defined conditions.
53	Ivy, J.L.; Goforth, H.W.; Damon, B.M.; McCauley, T.R.; Parsons, E.C.; Price, T.B. Early postexercise muscle glycogen recovery is enhanced with a carbohydrate-protein supplement. J. Appl. Physiol. 2002, 93, 1337-1344. https://doi.org/10.1152/japplphysiol.00314.2002	Human experimental study	Carbohydrate, glycogen, fluid, and sodium	EP/CDE; supports short-window glycogen restoration under defined conditions.
54	Pedersen, D.J.; Lessard, S.J.; Coffey, V.G.; Churchley, E.G.; Wootton, A.M.; Ng, T.; Watt, M.J.; Hawley, J.A. High rates of muscle glycogen resynthesis after exhaustive exercise when carbohydrate is coingested with caffeine. J. Appl. Physiol. 2008, 105, 7-13. https://doi.org/10.1152/japplphysiol.01121.2007	Human experimental study	Carbohydrate, glycogen, fluid, and sodium	EP/CDE; supports short-window glycogen restoration under defined conditions.
55	Sawka, M.N.; Burke, L.M.; Eichner, E.R.; Maughan, R.J.; Montain, S.J.; Stachenfeld, N.S. American College of Sports Medicine position stand. Exercise and fluid replacement. Med. Sci. Sports Exerc. 2007, 39, 377-390. https://doi.org/10.1249/mss.0b013e31802ca597	Position stand	Carbohydrate, glycogen, fluid, and sodium	EP or EP/CDE foundation; retained for accepted practice, safety, or methodological context.
56	McDermott, B.P.; Anderson, S.A.; Armstrong, L.E.; Casa, D.J.; Cheuvront, S.N.; Cooper, L.; Kenney, W.L.; O'Connor, F.G.; Roberts, W.O. National Athletic Trainers' Association position statement: Fluid replacement for the physically active. J. Athl. Train. 2017, 52, 877-895. https://doi.org/10.4085/1062-6050-52.9.02	Position statement	Carbohydrate, glycogen, fluid, and sodium	EP or EP/CDE foundation; retained for accepted practice, safety, or methodological context.
57	Nuccio, R.P.; Barnes, K.A.; Carter, J.M.; Baker, L.B. Fluid balance in team sport athletes and the effect of hypohydration on cognitive, technical, and physical performance. Sports Med. 2017, 47, 1951-1982. https://doi.org/10.1007/s40279-017-0738-7	Narrative review	Carbohydrate, glycogen, fluid, and sodium	EP or EP/CDE foundation; retained for accepted practice, safety, or methodological context.
58	Atherton, P.J.; Smith, K. Muscle protein synthesis in response to nutrition and exercise. J. Physiol. 2012, 590, 1049-1057. https://doi.org/10.1113/jphysiol.2011.225003	Mechanistic review	Protein quantity, timing, and remodeling	EP or EP/CDE foundation; retained for accepted practice, safety, or methodological context.
59	Morton, R.W.; Murphy, K.T.; McKellar, S.R.; Schoenfeld, B.J.; Henselmans, M.; Helms, E.; Aragon, A.A.; Devries, M.C.; Banfield, L.; Krieger, J.W.; et al. A systematic review, meta-analysis and meta-regression of the effect of protein supplementation on resistance training-induced gains in muscle mass and strength in healthy adults. Br. J. Sports Med. 2018, 52, 376-384. https://doi.org/10.1136/bjsports-2017-097608	Systematic review/meta-analysis/meta-regression	Protein quantity, timing, and remodeling	EP or EP/CDE foundation; retained for accepted practice, safety, or methodological context.
60	Phillips, S.M.; Van Loon, L.J.C. Dietary protein for athletes: From requirements to optimum adaptation. J. Sports Sci. 2011, 29, S29-S38. https://doi.org/10.1080/02640414.2011.619204	Narrative review	Protein quantity, timing, and remodeling	EP or EP/CDE foundation; retained for accepted practice, safety, or methodological context.
61	Jager, R.; Kerksick, C.M.; Campbell, B.I.; Cribb, P.J.; Wells, S.D.; Skwiat, T.M.; Purpura, M.; Ziegenfuss, T.N.; Ferrando, A.A.; Arent, S.M.; et al. International Society of Sports Nutrition position stand: Protein and exercise. J. Int. Soc. Sports Nutr. 2017, 14, 20. https://doi.org/10.1186/s12970-017-0177-8	Position stand	Protein quantity, timing, and remodeling	EP or EP/CDE foundation; retained for accepted practice, safety, or methodological context.
62	Areta, J.L.; Burke, L.M.; Ross, M.L.; Camera, D.M.; West, D.W.; Broad, E.M.; Jeacocke, N.A.; Moore, D.R.; Stellingwerff, T.; Phillips, S.M.; et al. Timing and distribution of protein ingestion during prolonged recovery from resistance exercise alters myofibrillar protein synthesis. J. Physiol. 2013, 591, 2319-2331. https://doi.org/10.1113/jphysiol.2012.244897	Randomized feeding study	Protein quantity, timing, and remodeling	EP/CDE; supports population- and context-bounded protein timing/dose statements.

Ref.	Full citation	Record type	Primary synthesis domain	Principal role / claim status
63	Moore, D.R.; Robinson, M.J.; Fry, J.L.; Tang, J.E.; Glover, E.I.; Wilkinson, S.B.; Prior, T.; Tarnopolsky, M.A.; Phillips, S.M. Ingested protein dose response of muscle and albumin protein synthesis after resistance exercise in young men. Am. J. Clin. Nutr. 2009, 89, 161-168. https://doi.org/10.3945/ajcn.2008.26401	Dose-response experiment	Protein quantity, timing, and remodeling	EP/CDE; supports population- and context-bounded protein timing/dose statements.
64	Witard, O.C.; Jackman, S.R.; Breen, L.; Smith, K.; Selby, A.; Tipton, K.D. Myofibrillar muscle protein synthesis rates subsequent to a meal in response to increasing doses of whey protein at rest and after resistance exercise. Am. J. Clin. Nutr. 2014, 99, 86-95. https://doi.org/10.3945/ajcn.112.055517	Dose-response experiment	Protein quantity, timing, and remodeling	EP/CDE; supports population- and context-bounded protein timing/dose statements.
65	Macnaughton, L.S.; Wardle, S.L.; Witard, O.C.; McGlory, C.; Hamilton, D.L.; Jeromson, S.; Lawrence, C.E.; Wallis, G.A.; Tipton, K.D. The response of muscle protein synthesis following whole-body resistance exercise is greater following 40 g than 20 g of ingested whey protein. Physiol. Rep. 2016, 4, e12893. https://doi.org/10.14814/phy2.12893	Randomized controlled trial	Protein quantity, timing, and remodeling	EP/CDE; supports population- and context-bounded protein timing/dose statements.
66	Tipton, K.D.; Rasmussen, B.B.; Miller, S.L.; Wolf, S.E.; Owens-Stovall, S.K.; Petrini, B.E.; Wolfe, R.R. Timing of amino acid-carbohydrate ingestion alters anabolic response of muscle to resistance exercise. Am. J. Physiol. Endocrinol. Metab. 2001, 281, E197-E206. https://doi.org/10.1152/ajpendo.2001.281.2.E197	Human experimental study	Protein quantity, timing, and remodeling	EP/CDE; supports population- and context-bounded protein timing/dose statements.
67	Biolo, G.; Tipton, K.D.; Klein, S.; Wolfe, R.R. An abundant supply of amino acids enhances the metabolic effect of exercise on muscle protein. Am. J. Physiol. 1997, 273, E122-E129. https://doi.org/10.1152/ajpendo.1997.273.1.E122	Human experimental study	Protein quantity, timing, and remodeling	EP/CDE; supports population- and context-bounded protein timing/dose statements.
68	Cermak, N.M.; Res, P.T.; de Groot, L.C.P.G.M.; Saris, W.H.M.; van Loon, L.J.C. Protein supplementation augments the adaptive response of skeletal muscle to resistance-type exercise training: A meta-analysis. Am. J. Clin. Nutr. 2012, 96, 1454-1464. https://doi.org/10.3945/ajcn.112.037556	Meta-analysis	Protein quantity, timing, and remodeling	EP or EP/CDE foundation; retained for accepted practice, safety, or methodological context.
69	Snijders, T.; Res, P.T.; Smeets, J.S.J.; van Vliet, S.; van Kranenburg, J.; Maase, K.; Kies, A.K.; Verdijk, L.B.; van Loon, L.J.C. Protein ingestion before sleep increases muscle mass and strength gains during prolonged resistance-type exercise training in healthy young men. J. Nutr. 2015, 145, 1178-1184. https://doi.org/10.3945/jn.114.208371	Randomized controlled trial	Protein quantity, timing, and remodeling	EP/CDE; supports population- and context-bounded protein timing/dose statements.
70	Res, P.T.; Groen, B.; Pennings, B.; Beelen, M.; Wallis, G.A.; Gijsen, A.P.; Senden, J.M.; van Loon, L.J.C. Protein ingestion before sleep improves postexercise overnight recovery. Med. Sci. Sports Exerc. 2012, 44, 1560-1569. https://doi.org/10.1249/MSS.0b013e31824cc363	Randomized controlled trial	Protein quantity, timing, and remodeling	EP/CDE; supports population- and context-bounded protein timing/dose statements.
71	Damas, F.; Phillips, S.M.; Libardi, C.A.; Vechin, F.C.; Lixandrao, M.E.; Jannig, P.R.; Costa, L.A.R.; Bacurau, A.V.; Snijders, T.; Parise, G.; et al. Resistance training-induced changes in integrated myofibrillar protein synthesis are related to hypertrophy only after attenuation of muscle damage. J. Physiol. 2016, 594, 5209-5222. https://doi.org/10.1113/JP272472	Longitudinal mechanistic training study	Protein quantity, timing, and remodeling	MR/CDE; separates repair-associated MPS from hypertrophy interpretation.
72	Shaw, G.; Lee-Barthel, A.; Ross, M.L.R.; Wang, B.; Baar, K. Vitamin C-enriched gelatin supplementation before intermittent activity augments collagen synthesis. Am. J. Clin. Nutr. 2017, 105, 136-143. https://doi.org/10.3945/ajcn.116.138594	Mechanistic crossover trial	Collagen/gelatin and connective tissue	MR/CDE; surrogate or pilot evidence only; no validated injury-prevention or return-to-play claim.
73	Lis, D.M.; Baar, K. Effects of different vitamin C-enriched collagen derivatives on collagen synthesis. Int. J. Sport Nutr. Exerc. Metab. 2019, 29, 526-531. https://doi.org/10.1123/ijsnem.2018-0385	Mechanistic intervention study	Collagen/gelatin and connective tissue	MR/CDE; surrogate or pilot evidence only; no validated injury-prevention or return-to-play claim.
74	Prowting, J.L.; Bemben, D.; Black, C.D.; Day, E.A.; Campbell, J.A. Effects of collagen peptides on recovery following eccentric exercise in resistance-trained males: A pilot study. Int. J. Sport Nutr. Exerc. Metab. 2021, 31, 32-39. https://doi.org/10.1123/IJSNEM.2020-0149	Pilot randomized study	Collagen/gelatin and connective tissue	MR/CDE; surrogate or pilot evidence only; no validated injury-prevention or return-to-play claim.
75	Owens, D.J.; Allison, R.; Close, G.L. Vitamin D and the athlete: Current perspectives and new challenges. Sports Med. 2018, 48, 3-16. https://doi.org/10.1007/s40279-017-0841-9	Narrative review	Vitamin D, energy availability, RED-S, and athlete nutrition	EP or EP/CDE foundation; retained for accepted practice, safety, or methodological context.
76	Farrokhyar, F.; Sivakumar, G.; Savage, K.; Koziarz, A.; Jamshidi, S.; Ayeni, O.R.; Peterson, D.; Bhandari, M. Effects of vitamin D supplementation on serum 25-hydroxyvitamin D concentrations and physical performance in athletes: A systematic review and meta-analysis of randomized controlled trials. Sports Med. 2017, 47, 2323-2339. https://doi.org/10.1007/s40279-017-0749-4	Systematic review and meta-analysis	Vitamin D, energy availability, RED-S, and athlete nutrition	EP or EP/CDE foundation; retained for accepted practice, safety, or methodological context.
77	Close, G.L.; Russell, J.; Cobley, J.N.; Owens, D.J.; Wilson, G.; Gregson, W.; Fraser, W.D.; Morton, J.P. Assessment of vitamin D concentration in non-supplemented professional athletes and healthy adults during the winter months in the UK: Implications for skeletal muscle function. J. Sports Sci. 2013, 31, 344-353. https://doi.org/10.1080/02640414.2012.733822	Observational study	Vitamin D, energy availability, RED-S, and athlete nutrition	Contextual support within the narrative synthesis.
78	Mountjoy, M.; Sundgot-Borgen, J.; Burke, L.; Carter, S.; Constantini, N.; Lebrun, C.; Meyer, N.; Sherman, R.; Steffen, K.; Budgett, R.; et al. The IOC consensus statement: Beyond the Female Athlete Triad--Relative Energy Deficiency in Sport (RED-S). Br. J. Sports Med. 2014, 48, 491-497. https://doi.org/10.1136/bjsports-2014-093502	Consensus statement	Vitamin D, energy availability, RED-S, and athlete nutrition	EP or EP/CDE foundation; retained for accepted practice, safety, or methodological context.
79	Mountjoy, M.; Sundgot-Borgen, J.; Burke, L.; Ackerman, K.E.; Blauwet, C.; Constantini, N.; Lebrun, C.; Lundy, B.; Melin, A.K.; Meyer, N.L.; et al. IOC consensus statement on relative energy deficiency in sport (RED-S): 2018 update. Br. J. Sports Med. 2018, 52, 687-697. https://doi.org/10.1136/bjsports-2018-099193	Consensus statement	Vitamin D, energy availability, RED-S, and athlete nutrition	EP or EP/CDE foundation; retained for accepted practice, safety, or methodological context.
80	Loucks, A.B.; Kiens, B.; Wright, H.H. Energy availability in athletes. J. Sports Sci. 2011, 29, S7-S15. https://doi.org/10.1080/02640414.2011.588958	Narrative review	Vitamin D, energy availability, RED-S, and athlete nutrition	EP or EP/CDE foundation; retained for accepted practice, safety, or methodological context.
81	Burke, L.M.; Castell, L.M.; Casa, D.J.; Close, G.L.; Costa, R.J.S.; Desbrow, B.; Halson, S.L.; Lis, D.M.; Melin, A.K.; Peeling, P.; et al. International Association of Athletics Federations consensus statement 2019: Nutrition for athletics. Int. J. Sport Nutr. Exerc. Metab. 2019, 29, 73-84. https://doi.org/10.1123/ijsnem.2019-0065	Consensus statement	Vitamin D, energy availability, RED-S, and athlete nutrition	EP or EP/CDE foundation; retained for accepted practice, safety, or methodological context.
82	Halson, S.L. Sleep in elite athletes and nutritional interventions to enhance sleep. Sports Med. 2014, 44, S13-S23. https://doi.org/10.1007/s40279-014-0147-0	Narrative review	Sleep, load, monitoring, and in-season recovery	EP or EP/CDE foundation; retained for accepted practice, safety, or methodological context.

Ref.	Full citation	Record type	Primary synthesis domain	Principal role / claim status
83	Fullagar, H.H.K.; Skorski, S.; Duffield, R.; Hammes, D.; Coutts, A.J.; Meyer, T. Sleep and athletic performance: The effects of sleep loss on exercise performance, and physiological and cognitive responses to exercise. Sports Med. 2015, 45, 161-186. https://doi.org/10.1007/s40279-014-0260-0	Narrative review	Sleep, load, monitoring, and in-season recovery	EP or EP/CDE foundation; retained for accepted practice, safety, or methodological context.
84	Bonnar, D.; Bartel, K.; Kakoschke, N.; Lang, C. Sleep interventions designed to improve athletic performance and recovery: A systematic review of current approaches. Sports Med. 2018, 48, 683-703. https://doi.org/10.1007/s40279-017-0832-x	Systematic review	Sleep, load, monitoring, and in-season recovery	EP or EP/CDE foundation; retained for accepted practice, safety, or methodological context.
85	Halson, S.L. Monitoring training load to understand fatigue in athletes. Sports Med. 2014, 44, S139-S147. https://doi.org/10.1007/s40279-014-0253-z	Narrative review	Sleep, load, monitoring, and in-season recovery	EP or EP/CDE foundation; retained for accepted practice, safety, or methodological context.
86	Bourdon, P.C.; Cardinale, M.; Murray, A.; Gastin, P.; Kellmann, M.; Varley, M.C.; Gabbett, T.J.; Coutts, A.J.; Burgess, D.J.; Gregson, W.; et al. Monitoring athlete training loads: Consensus statement. Int. J. Sports Physiol. Perform. 2017, 12, S2-161-S2-170. https://doi.org/10.1123/IJSPP.2017-0208	Consensus statement	Sleep, load, monitoring, and in-season recovery	EP or EP/CDE foundation; retained for accepted practice, safety, or methodological context.
87	Saw, A.E.; Main, L.C.; Gastin, P.B. Monitoring the athlete training response: Subjective self-reported measures trump commonly used objective measures: A systematic review. Br. J. Sports Med. 2016, 50, 281-291. https://doi.org/10.1136/bjsports-2015-094758	Systematic review	Sleep, load, monitoring, and in-season recovery	EP or EP/CDE foundation; retained for accepted practice, safety, or methodological context.
88	Buchheit, M. Monitoring training status with heart rate measures: Do all roads lead to Rome? Front. Physiol. 2014, 5, 73. https://doi.org/10.3389/fphys.2014.00073	Narrative review	Sleep, load, monitoring, and in-season recovery	EP or EP/CDE foundation; retained for accepted practice, safety, or methodological context.
89	Meeusen, R.; Duclos, M.; Foster, C.; Fry, A.; Gleeson, M.; Nieman, D.; Raglin, J.; Rietjens, G.; Steinacker, J.; Urhausen, A. Prevention, diagnosis and treatment of the overtraining syndrome: Joint consensus statement. Med. Sci. Sports Exerc. 2013, 45, 186-205. https://doi.org/10.1249/MSS.0b013e318279a10a	Consensus statement	Sleep, load, monitoring, and in-season recovery	EP or EP/CDE foundation; retained for accepted practice, safety, or methodological context.
90	Impellizzeri, F.M.; Marcora, S.M.; Coutts, A.J. Internal and external training load: 15 years on. Int. J. Sports Physiol. Perform. 2019, 14, 270-273. https://doi.org/10.1123/ijspp.2018-0935	Methodological commentary	Sleep, load, monitoring, and in-season recovery	EP or EP/CDE foundation; retained for accepted practice, safety, or methodological context.
91	Foster, C.; Florhaug, J.A.; Franklin, J.; Gottschall, L.; Hrovatin, L.A.; Parker, S.; Doleshal, P.; Dodge, C. A new approach to monitoring exercise training. J. Strength Cond. Res. 2001, 15, 109-115. https://doi.org/10.1519/00124278-200102000-00019	Monitoring method study	Sleep, load, monitoring, and in-season recovery	EP or EP/CDE foundation; retained for accepted practice, safety, or methodological context.
92	Heaton, L.E.; Davis, J.K.; Rawson, E.S.; Nuccio, R.P.; Witard, O.C.; Stein, K.W.; Baar, K.; Carter, J.M.; Baker, L.B. Selected in-season nutritional strategies to enhance recovery for team sport athletes: A practical overview. Sports Med. 2017, 47, 2201-2218. https://doi.org/10.1007/s40279-017-0759-2	Practical review	Sleep, load, monitoring, and in-season recovery	EP or EP/CDE foundation; retained for accepted practice, safety, or methodological context.
93	Jones, A.M. Dietary nitrate supplementation and exercise performance. Sports Med. 2014, 44, S35-S45. https://doi.org/10.1007/s40279-014-0149-y	Narrative review	Nitrate, creatine, EIMD, and polyphenol synthesis	EP for selected performance outcomes; not treated as established EIMD-recovery evidence.
94	Bailey, S.J.; Winyard, P.; Vanhatalo, A.; Blackwell, J.R.; DiMenna, F.J.; Wilkerson, D.P.; Tarr, J.; Benjamin, N.; Jones, A.M. Dietary nitrate supplementation reduces the O2 cost of low-intensity exercise and enhances tolerance to high-intensity exercise in humans. J. Appl. Physiol. 2009, 107, 1144-1155. https://doi.org/10.1152/japplphysiol.00722.2009	Randomized controlled trial	Nitrate, creatine, EIMD, and polyphenol synthesis	EP for selected performance outcomes; not treated as established EIMD-recovery evidence.
95	Lansley, K.E.; Winyard, P.G.; Fulford, J.; Vanhatalo, A.; Bailey, S.J.; Blackwell, J.R.; DiMenna, F.J.; Gilchrist, M.; Benjamin, N.; Jones, A.M. Dietary nitrate supplementation reduces the O2 cost of walking and running: A placebo-controlled study. J. Appl. Physiol. 2011, 110, 591-600. https://doi.org/10.1152/japplphysiol.01070.2010	Randomized controlled trial	Nitrate, creatine, EIMD, and polyphenol synthesis	EP for selected performance outcomes; not treated as established EIMD-recovery evidence.
96	Kreider, R.B.; Kalman, D.S.; Antonio, J.; Ziegenfuss, T.N.; Wildman, R.; Collins, R.; Candow, D.G.; Kleiner, S.M.; Almada, A.L.; Lopez, H.L. International Society of Sports Nutrition position stand: Safety and efficacy of creatine supplementation in exercise, sport, and medicine. J. Int. Soc. Sports Nutr. 2017, 14, 18. https://doi.org/10.1186/s12970-017-0173-z	Position stand	Nitrate, creatine, EIMD, and polyphenol synthesis	EP or EP/CDE foundation; retained for accepted practice, safety, or methodological context.
97	Cooke, M.B.; Rybalka, E.; Williams, A.D.; Cribb, P.J.; Hayes, A. Creatine supplementation enhances muscle force recovery after eccentrically-induced muscle damage in healthy individuals. J. Int. Soc. Sports Nutr. 2009, 6, 13. https://doi.org/10.1186/1550-2783-6-13	Randomized controlled trial	Nitrate, creatine, EIMD, and polyphenol synthesis	CDE; recovery-specific effects remain heterogeneous or product-specific.
98	Branch, J.D. Effect of creatine supplementation on body composition and performance: A meta-analysis. Int. J. Sport Nutr. Exerc. Metab. 2003, 13, 198-226. https://doi.org/10.1123/ijsnem.13.2.198	Meta-analysis	Nitrate, creatine, EIMD, and polyphenol synthesis	EP or EP/CDE foundation; retained for accepted practice, safety, or methodological context.
99	Northeast, B.; Clifford, T. The effect of creatine supplementation on markers of exercise-induced muscle damage: A systematic review and meta-analysis of human intervention trials. Int. J. Sport Nutr. Exerc. Metab. 2021, 31, 276-291. https://doi.org/10.1123/ijsnem.2020-0282	Systematic review and meta-analysis	Nitrate, creatine, EIMD, and polyphenol synthesis	CDE; recovery-specific effects remain heterogeneous or product-specific.
100	Harty, P.S.; Cottet, M.L.; Malloy, J.K.; Kerksick, C.M. Nutritional and supplementation strategies to prevent and attenuate exercise-induced muscle damage: A brief review. Sports Med. Open 2019, 5, 1. https://doi.org/10.1186/s40798-018-0176-6	Narrative review	Nitrate, creatine, EIMD, and polyphenol synthesis	CDE; recovery-specific effects remain heterogeneous or product-specific.
101	Rickards, L.; Lynn, A.; Harrop, D.; Barker, M.E.; Russell, M.; Ranchordas, M.K. Effect of polyphenol-rich foods, juices, and concentrates on recovery from exercise-induced muscle damage: A systematic review and meta-analysis. Nutrients 2021, 13, 2988. https://doi.org/10.3390/nu13092988	Systematic review and meta-analysis	Nitrate, creatine, EIMD, and polyphenol synthesis	CDE; recovery-specific effects remain heterogeneous or product-specific.
102	Sim, M.; Garvican-Lewis, L.A.; Cox, G.R.; Govus, A.; McKay, A.K.A.; Stellingwerff, T.; Peeling, P. Iron considerations for the athlete: A narrative review. Eur. J. Appl. Physiol. 2019, 119, 1463-1478. https://doi.org/10.1007/s00421-019-04157-y	Narrative review	Iron, magnesium, RED-S, and caffeine	EP or EP/CDE foundation; retained for accepted practice, safety, or methodological context.
103	Peeling, P.; Dawson, B.; Goodman, C.; Landers, G.; Trinder, D. Athletic induced iron deficiency: New insights into the role of inflammation, cytokines and hormones. Eur. J. Appl. Physiol. 2008, 103, 381-391. https://doi.org/10.1007/s00421-008-0726-6	Narrative review	Iron, magnesium, RED-S, and caffeine	EP or EP/CDE foundation; retained for accepted practice, safety, or methodological context.

Ref.	Full citation	Record type	Primary synthesis domain	Principal role / claim status
104	Volpe, S.L. Magnesium and the athlete. Curr. Sports Med. Rep. 2015, 14, 279-283. https://doi.org/10.1249/JSR.0000000000000178	Narrative review	Iron, magnesium, RED-S, and caffeine	EP or EP/CDE foundation; retained for accepted practice, safety, or methodological context.
105	Zhang, Y.; Xun, P.; Wang, R.; Mao, L.; He, K. Can magnesium enhance exercise performance? Nutrients 2017, 9, 946. https://doi.org/10.3390/nu9090946	Narrative review	Iron, magnesium, RED-S, and caffeine	EP or EP/CDE foundation; retained for accepted practice, safety, or methodological context.
106	Mountjoy, M.; Ackerman, K.E.; Bailey, D.M.; Burke, L.M.; Constantini, N.; Hackney, A.C.; Heikura, I.A.; Melin, A.; Pensgaard, A.M.; Stellingwerff, T.; Sundgot-Borgen, J.K.; et al. 2023 International Olympic Committee's (IOC) consensus statement on Relative Energy Deficiency in Sport (REDs). Br. J. Sports Med. 2023, 57, 1073-1097. https://doi.org/10.1136/bjsports-2023-106994	Consensus statement	Iron, magnesium, RED-S, and caffeine	EP or EP/CDE foundation; retained for accepted practice, safety, or methodological context.
107	Guest, N.S.; VanDusseldorp, T.A.; Nelson, M.T.; Grgic, J.; Schoenfeld, B.J.; Jenkins, N.D.M.; Arent, S.M.; Antonio, J.; Stout, J.R.; Trexler, E.T.; et al. International Society of Sports Nutrition position stand: Caffeine and exercise performance. J. Int. Soc. Sports Nutr. 2021, 18, 1. https://doi.org/10.1186/s12970-020-00383-4	Position stand	Iron, magnesium, RED-S, and caffeine	EP or EP/CDE foundation; retained for accepted practice, safety, or methodological context.
108	Brancaccio, P.; Maffulli, N.; Limongelli, F.M. Creatine kinase monitoring in sport medicine. Br. Med. Bull. 2007, 81-82, 209-230. https://doi.org/10.1093/bmb/ldm014	Narrative review	Biomarkers, recovery modalities, and monitoring	EP or EP/CDE foundation; retained for accepted practice, safety, or methodological context.
109	Lee, E.C.; Fragala, M.S.; Kavouras, S.A.; Queen, R.M.; Pryor, J.L.; Casa, D.J. Biomarkers in sports and exercise: Tracking health, performance, and recovery in athletes. J. Strength Cond. Res. 2017, 31, 2920-2937. https://doi.org/10.1519/JSC.0000000000002122	Narrative review	Biomarkers, recovery modalities, and monitoring	EP or EP/CDE foundation; retained for accepted practice, safety, or methodological context.
110	Dupuy, O.; Douzi, W.; Theurot, D.; Bosquet, L.; Dugue, B. An evidence-based approach for choosing post-exercise recovery techniques to reduce markers of muscle damage, soreness, fatigue, and inflammation: A systematic review with meta-analysis. Front. Physiol. 2018, 9, 403. https://doi.org/10.3389/fphys.2018.00403	Systematic review and meta-analysis	Biomarkers, recovery modalities, and monitoring	CDE; context for recovery modalities and symptom outcomes, not RAC validation.
111	Machado, A.F.; Ferreira, P.H.; Micheletti, J.K.; de Almeida, A.C.; Lemes, I.R.; Vanderlei, F.M.; Netto Junior, J.; Pastre, C.M. Can water temperature and immersion time influence the effect of cold-water immersion on muscle soreness? A systematic review and meta-analysis. Sports Med. 2016, 46, 503-514. https://doi.org/10.1007/s40279-015-0431-7	Systematic review and meta-analysis	Biomarkers, recovery modalities, and monitoring	CDE; context for recovery modalities and symptom outcomes, not RAC validation.
112	Mănescu, D.C.; Voinea, A.; Plastoi, C.D.; Iacobini, A.R.; Vulpe, A.A.; Pîrvan, A.; Dinciu, C.C.; Vulpe, B.I.; Băltărețu, C.; Iacobini, A. Molecular Biomarkers of Training Responses: A Systems Framework for Exercise Adaptation and Athlete Monitoring. Int. J. Mol. Sci. 2026, 27, 3601. https://doi.org/10.3390/ijms27083601	Author-group conceptual framework	Biomarkers, recovery modalities, and monitoring	RH positioning only; not used as empirical validation or intervention-efficacy evidence.
113	McNulty, K.L.; Elliott-Sale, K.J.; Dolan, E.; Swinton, P.A.; Ansdell, P.; Goodall, S.; Thomas, K.; Hicks, K.M. The effects of menstrual cycle phase on exercise performance in eumenorrheic women: A systematic review and meta-analysis. Sports Med. 2020, 50, 1813-1827. https://doi.org/10.1007/s40279-020-01319-3	Systematic review and meta-analysis	Female-athlete and menstrual-cycle evidence	CDE/MR; informs contextual variables and research gaps, not deterministic prescriptions.
114	Elliott-Sale, K.J.; Minahan, C.L.; de Jonge, X.A.K.J.; Ackerman, K.E.; Sipila, S.; Constantini, N.W.; Lebrun, C.M.; Hackney, A.C. Methodological considerations for studies in sport and exercise science with women as participants: A working guide for standards of practice for research on women. Sports Med. 2021, 51, 843-861. https://doi.org/10.1007/s40279-021-01435-8	Methodological guide	Female-athlete and menstrual-cycle evidence	CDE/MR; informs contextual variables and research gaps, not deterministic prescriptions.
115	Romero-Parra, N.; Cupeiro, R.; Alfaro-Magallanes, V.M.; Rael, B.; Rubio-Arias, J.A.; Peinado, A.B.; Benito, P.J.; IronFEMME Study Group. Exercise-induced muscle damage during the menstrual cycle: A systematic review and meta-analysis. J. Strength Cond. Res. 2021, 35, 549-561. https://doi.org/10.1519/JSC.0000000000003878	Systematic review and meta-analysis	Female-athlete and menstrual-cycle evidence	CDE/MR; informs contextual variables and research gaps, not deterministic prescriptions.
116	Clark, A.; Mach, N. Exercise-induced stress behavior, gut-microbiota-brain axis and diet: A systematic review for athletes. J. Int. Soc. Sports Nutr. 2016, 13, 43. https://doi.org/10.1186/s12970-016-0155-6	Systematic review	Gut microbiome and gastrointestinal context	CDE/MR; informs contextual variables and research gaps, not deterministic prescriptions.
117	Jager, R.; Mohr, A.E.; Carpenter, K.C.; Kerksick, C.M.; Purpura, M.; Moussa, A.; Townsend, J.R.; West, N.P.; Black, K.; Gleeson, M.; et al. International Society of Sports Nutrition position stand: Probiotics. J. Int. Soc. Sports Nutr. 2019, 16, 62. https://doi.org/10.1186/s12970-019-0329-0	Position stand	Gut microbiome and gastrointestinal context	CDE/MR; informs contextual variables and research gaps, not deterministic prescriptions.
118	Mohr, A.E.; Jager, R.; Carpenter, K.C.; Kerksick, C.M.; Purpura, M.; Townsend, J.R.; West, N.P.; Black, K.; Gleeson, M.; Pyne, D.B.; et al. The athletic gut microbiota. J. Int. Soc. Sports Nutr. 2020, 17, 24. https://doi.org/10.1186/s12970-020-00353-w	Narrative review	Gut microbiome and gastrointestinal context	CDE/MR; informs contextual variables and research gaps, not deterministic prescriptions.
119	Mailing, L.J.; Allen, J.M.; Buford, T.W.; Fields, C.J.; Woods, J.A. Exercise and the gut microbiome: A review of the evidence, potential mechanisms, and implications for human health. Exerc. Sport Sci. Rev. 2019, 47, 75-85. https://doi.org/10.1249/JES.0000000000000183	Narrative review	Gut microbiome and gastrointestinal context	CDE/MR; informs contextual variables and research gaps, not deterministic prescriptions.
120	Monda, V.; Villano, I.; Messina, A.; Valenzano, A.; Esposito, T.; Moscatelli, F.; Viggiano, A.; Cibelli, G.; Chieffi, S.; Monda, M.; et al. Exercise modifies the gut microbiota with positive health effects. Oxid. Med. Cell. Longev. 2017, 2017, 3831972. https://doi.org/10.1155/2017/3831972	Narrative review	Gut microbiome and gastrointestinal context	CDE/MR; informs contextual variables and research gaps, not deterministic prescriptions.
121	Ribas, M.R.; Schneider, F.K.; Ribas, D.I.R.; Badicu, G.; Bonatto, A.C.; Ardigò, L.P.; Bassan, J.C. Impact of Genetic Polymorphisms on Electrochemical Parameters and Acid-Base Disorders in Brazilian Runners During a 105-Kilometer Ultramarathon. Nutrients 2024, 16, 3945. https://doi.org/10.3390/nu16223945	Observational field/genotype-association study	Individual variability, electrochemical and acid–base responses, ultra-endurance monitoring	CDE; illustrates genotype-associated variability in electrochemical and acid–base responses during extreme endurance exercise.
122	Gibala, M.J.; Little, J.P.; Macdonald, M.J.; Hawley, J.A. Physiological adaptations to low-volume, high-intensity interval training in health and disease. J. Physiol. 2012, 590, 1077-1084. https://doi.org/10.1113/jphysiol.2011.224725	Narrative review	Molecular adaptation and concurrent training	MR; molecular pathway or training-interference context, not direct RAC validation.
123	Fyfe, J.J.; Bishop, D.J.; Stepto, N.K. Interference between concurrent resistance and endurance exercise: Molecular bases and the role of individual training variables. Sports Med. 2014, 44, 743-762. https://doi.org/10.1007/s40279-014-0162-1	Narrative review	Molecular adaptation and concurrent training	MR; molecular pathway or training-interference context, not direct RAC validation.

Ref.	Full citation	Record type	Primary synthesis domain	Principal role / claim status
124	Hardie, D.G.; Ross, F.A.; Hawley, S.A. AMPK: A nutrient and energy sensor that maintains energy homeostasis. Nat. Rev. Mol. Cell Biol. 2012, 13, 251-262. https://doi.org/10.1038/nrm3311	Mechanistic review	Molecular adaptation and concurrent training	MR; molecular pathway or training-interference context, not direct RAC validation.
125	Laplante, M.; Sabatini, D.M. mTOR signaling in growth control and disease. Cell 2012, 149, 274-293. https://doi.org/10.1016/j.cell.2012.03.017	Mechanistic review	Molecular adaptation and concurrent training	MR; molecular pathway or training-interference context, not direct RAC validation.
126	Canto, C.; Auwerx, J. PGC-1alpha, SIRT1 and AMPK, an energy sensing network that controls energy expenditure. Curr. Opin. Lipidol. 2009, 20, 98-105. https://doi.org/10.1097/MOL.0b013e328328d0a4	Mechanistic review	Molecular adaptation and concurrent training	MR; molecular pathway or training-interference context, not direct RAC validation.
127	Baar, K. Using molecular biology to maximize concurrent training. Sports Med. 2014, 44, S117-S125. https://doi.org/10.1007/s40279-014-0252-0	Mechanistic review	Molecular adaptation and concurrent training	MR; molecular pathway or training-interference context, not direct RAC validation.
128	Trexler, E.T.; Smith-Ryan, A.E.; Stout, J.R.; Hoffman, J.R.; Wilborn, C.D.; Sale, C.; Kreider, R.B.; Jager, R.; Earnest, C.P.; Bannock, L.; et al. International Society of Sports Nutrition position stand: Beta-alanine. J. Int. Soc. Sports Nutr. 2015, 12, 30. https://doi.org/10.1186/s12970-015-0090-y	Position stand	Supplement efficacy and safety positions	EP or EP/CDE foundation; retained for accepted practice, safety, or methodological context.
129	Grgic, J.; Pedisic, Z.; Saunders, B.; Artioli, G.G.; Schoenfeld, B.J.; McKenna, M.J.; Bishop, D.J.; Kreider, R.B.; Stout, J.R.; Kalman, D.S.; et al. International Society of Sports Nutrition position stand: Sodium bicarbonate and exercise performance. J. Int. Soc. Sports Nutr. 2021, 18, 61. https://doi.org/10.1186/s12970-021-00458-w	Position stand	Supplement efficacy and safety positions	EP or EP/CDE foundation; retained for accepted practice, safety, or methodological context.
130	Wilson, J.M.; Fitschen, P.J.; Campbell, B.; Wilson, G.J.; Zanchi, N.; Taylor, L.; Wilborn, C.; Kalman, D.S.; Stout, J.R.; Hoffman, J.R.; et al. International Society of Sports Nutrition position stand: Beta-hydroxy-beta-methylbutyrate (HMB). J. Int. Soc. Sports Nutr. 2013, 10, 6. https://doi.org/10.1186/1550-2783-10-6	Position stand	Supplement efficacy and safety positions	EP or EP/CDE foundation; retained for accepted practice, safety, or methodological context.

Inventory note. References 12–16 and 112 (6/130; 4.6%) include one or more members of the current author group and are explicitly restricted to conceptual positioning or biomarker-organization context. They are not used as empirical validation of RAC or as intervention-efficacy evidence.
